# Normal saline remodels the omentum and stimulates its receptivity for transcoelomic metastasis

**DOI:** 10.1172/jci.insight.167336

**Published:** 2023-06-22

**Authors:** Hironari Akasaka, WonJae Lee, Song Yi Ko, Ernst Lengyel, Honami Naora

**Affiliations:** 1 Department of Molecular and Cellular Oncology, University of Texas MD Anderson Cancer Center, Houston, Texas, USA.; 2 Department of Obstetrics and Gynecology, Section of Gynecologic Oncology, University of Chicago, Chicago, Illinois, USA.

**Keywords:** Oncology, Adipose tissue, Cancer, Macrophages

## Abstract

The omentum contains immune cell structures called milky spots that are niches for transcoelomic metastasis. It is difficult to remove the omentum completely, and there are no effective strategies to minimize the risk of colonization of preserved omental tissues by cancer cells that circulate in the peritoneal fluid. Normal saline is commonly administered into the peritoneal cavity for diagnostic and intraoperative lavage. Here we show that normal saline, when administered into the peritoneal cavity of mice, is prominently absorbed by the omentum, exfoliates its mesothelium, and induces expression of CX3CL1, the ligand for CX3CR1, within and surrounding the omental vasculature. Studies using CX3CR1-competent and CX3CR1-deficient mice showed that the predominant response in the omentum following saline administration is an accumulation of CX3CR1^+^ monocytes/macrophages that expand milky spots and promote neoangiogenesis within these niches. Moreover, saline administration promoted the implantation of cancer cells of ovarian and colorectal origin onto the omentum. By contrast, these deleterious effects were not observed following i.p. administration of lactated Ringer’s solution. Our findings suggest that normal saline stimulates the receptivity of the omentum for cancer cells and that the risk of colonization can be minimized by using a biocompatible crystalloid for lavage procedures.

## Graphical abstract

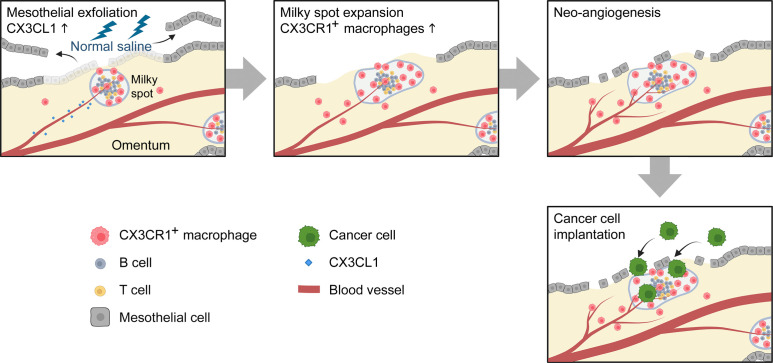

## Introduction

The omentum comprises 2 structures that extend from the greater and lesser curvatures of the stomach, respectively, and are mostly composed of adipocytes with a protective mesothelial lining (1). Although commonly termed an adipose tissue, the omentum is distinguished from other visceral fat depots by several striking properties. The omentum contains an extensive vascular and lymphatic network and plays an important function in peritoneal homeostasis by absorbing fluid from the peritoneal cavity (2). Furthermore, the omentum is characterized by its abundance of immune cell structures called milky spots that concentrate and eliminate pathogens from the peritoneal cavity (1, 3). In reference to its central role in peritoneal defense, the omentum was described more than 100 years ago as a “sponge” for septic material (4) and as the “abdominal policeman” (5).

The absorptive and filtering properties of the omentum are problematic in malignancy, as cancer cells that circulate in the peritoneal fluid can be trapped in milky spots and form implants (6, 7). Transcoelomic metastasis to the omentum occurs in gastrointestinal cancers and almost invariably in advanced-stage ovarian cancer (8, 9). Growth of tumor implants on the omentum angulates the bowel, causing obstruction and substantial pain. Although the diseased tissue is removed in almost all cases, it is difficult to completely resect the omentum because of its anatomic location that reaches to the splenic hilum. Furthermore, there is a lack of consensus as to the amount of healthy omental tissue that should be removed in patients who present without overt metastasis. One school of thought has advocated omental biopsies rather than removal of the omentum in these cases, as omentectomy may have complications such as increasing the risk of sepsis, adhesive bowel obstruction, and splenic injury (10–14). In support of this line of thinking, a study using a rat model of ovarian cancer found that prophylactic omentectomy did not improve survival (15). In addition, a study of Surveillance, Epidemiology, and End Results data found that omentectomy did not improve survival of women with Stage I/II ovarian cancer (i.e., with ovarian-confined or locally extended disease) (16). Other studies have also supported the preservation of omental tissue in patients with early-stage gastric and endometrial cancers (17, 18).

A limitation to preserving healthy omental tissue is the risk of its colonization by occult cancer cells that circulate in the peritoneal fluid (9). We therefore considered whether the risk of omental colonization might be minimized by modifying other practices. Normal saline (0.9% sodium chloride) is the most widely utilized crystalloid for lavage and is administered into the peritoneal cavity for diagnostic and intraoperative washings (19, 20). Normal saline has a similar osmolality to body fluids but has a pH of approximately 5.5 (21). The only well-documented deleterious effect of normal saline in the peritoneal cavity is adhesion formation (22–24). It has been estimated that 25% of instilled normal saline is not drained but dwells in the cavity (24). Because of the absorptive ability of the omentum, we hypothesized that this tissue might be exquisitely sensitive to normal saline and elicit unique responses. Here we show that normal saline, when administered i.p. to mice, is prominently absorbed by the omentum, stimulates the expansion of milky spots and neoangiogenesis through the recruitment of CX3CR1^+^ monocyte/macrophages, and promotes implantation of cancer cells onto the omentum. By contrast, these effects were not observed following i.p. administration of lactated Ringer’s solution (LRS), a balanced crystalloid. Our findings implicate that normal saline “primes” the omentum for implantation of cancer cells and that the risk of colonization can be minimized by using a more biocompatible solution in lavage procedures.

## Results

### Normal saline is prominently absorbed by the omentum and exfoliates its mesothelium.

**Figure 1:**
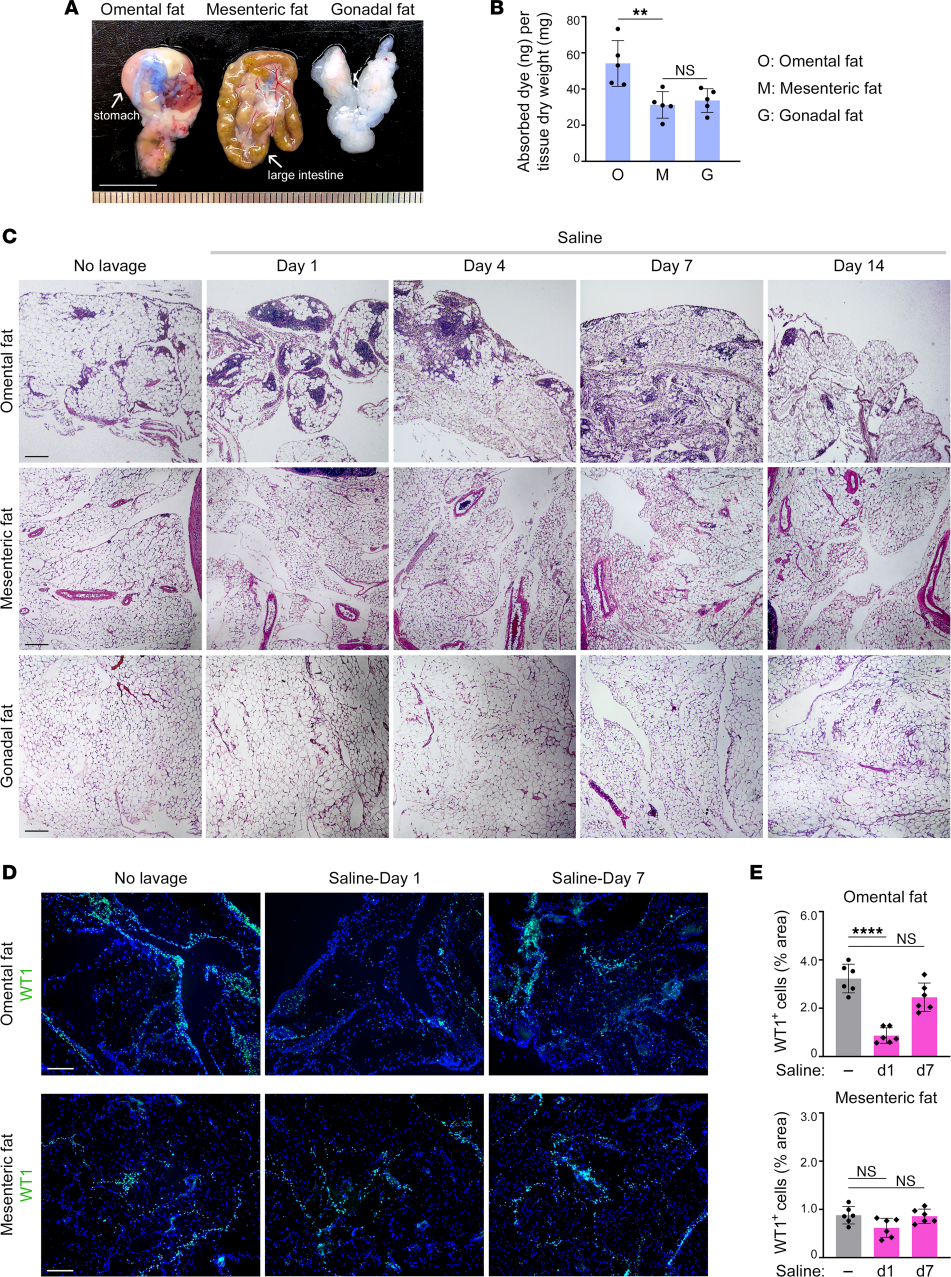
Normal saline is prominently absorbed by the omentum and exfoliates its mesothelium. (**A** and **B**) Absorption of normal saline by peritoneal fat tissues. (**A**) Representative images of omental, mesenteric, and gonadal fat tissues at 1 hour following i.p. administration of normal saline (12.5 mL/kg) containing Evans Blue dye to mice. Scale bar: 10 mm. (**B**) Quantification of dye in each tissue. Data of *n* = 5 mice are shown. (**C**) Representative images of H&E-stained sections of peritoneal fat tissues of untreated mice and mice at the indicated time points following i.p. administration of normal saline. Scale bar: 200 μm. Tissues of *n* = 6 mice per group were evaluated. (**D** and **E**) WT1^+^ cells in peritoneal fat tissues of untreated mice and mice at day 1 and day 7 following saline administration (*n* = 6 per group). (**D**) Representative images of immunofluorescence staining of WT1 (shown in green). Tissues were counterstained with DAPI. Scale bar: 200 μm. (**E**) Abundance of WT1^+^ cells, expressed as the percentage of area within each tissue. An average score for each tissue of each mouse was calculated by evaluating WT1 staining in 4–5 random and independent 40***×*** microscopic fields. Adult female C57BL/6 mice were used in **A**–**E**. **P < 0.01, ****P < 0.0001, by Tukey’s multiple comparisons test in **B** and by Dunnett’s multiple comparisons test compared with untreated mice in **E**.

In this study, we administered normal saline i.p. to adult female C57BL/6 mice at 12.5 mL/kg body weight. This dose was based on the administration of 1 liter of saline to an adult female with an average body weight of 80 kg. Typically, 1 liter is administered for diagnostic lavage and larger volumes are used intraoperatively (19). As reported in other rodent studies (25), we found that the baseline pH in the peritoneal cavity of mice is slightly lower than in humans. Following i.p. administration of normal saline, peritoneal pH decreased over 24 hours and thereafter regained the baseline level (Supplemental Figure 1, A and B; supplemental material available online with this article; https://doi.org/10.1172/jci.insight.167336DS1). To evaluate fluid absorption, normal saline containing vessel-tracing Evans Blue dye was administered i.p. The most striking absorption was observed in the omentum as compared with mesenteric and gonadal fat depots (Figure 1, A and B).

H&E-stained sections of omental tissues showed that nonadipocyte cellularity was increased from day 1 to day 7 following administration of normal saline, and returned to the baseline level by day 14 (Figure 1C). By contrast, no histologic changes were observed in mesenteric and gonadal fat tissues (Figure 1C). To evaluate mesothelial linings of serosal surfaces, we stained for Wilms tumor protein (WT1). Following saline administration, omental surfaces were denuded of WT1^+^ cells at day 1 (*P* < 0.0001), and by day 7, the mesothelium had largely regenerated (Figure 1, D and E). By contrast, the abundance of WT1^+^ cells in the mesentery did not significantly change (Figure 1, D and E). To confirm these findings, mesothelial cells were quantified by flow cytometry. Mesothelial cells express podoplanin (PDPN) and, unlike fibroblasts, lack CD140a (platelet-derived growth factor receptor-α) (26). Gating strategy and confirmation that the vast majority (~95%) of CD45^–^PDPN^+^CD140a^–^ cells are WT1^+^ are shown in Supplemental Figure 2, A and B. Following saline administration, numbers of CD45^–^PDPN^+^CD140a^–^ cells decreased in the omentum at day 1 (*P* < 0.01) and were mostly restored at day 7, whereas no significant changes were detected in mesenteric and gonadal fat tissues (Supplemental Figure 2C). These findings indicate that the omentum, as compared with other peritoneal fat depots, highly absorbs normal saline and undergoes extensive mesothelial cell exfoliation and tissue remodeling.

### Normal saline stimulates transient expansion of milky spots and neoangiogenesis in the omentum.

**Figure 2:**
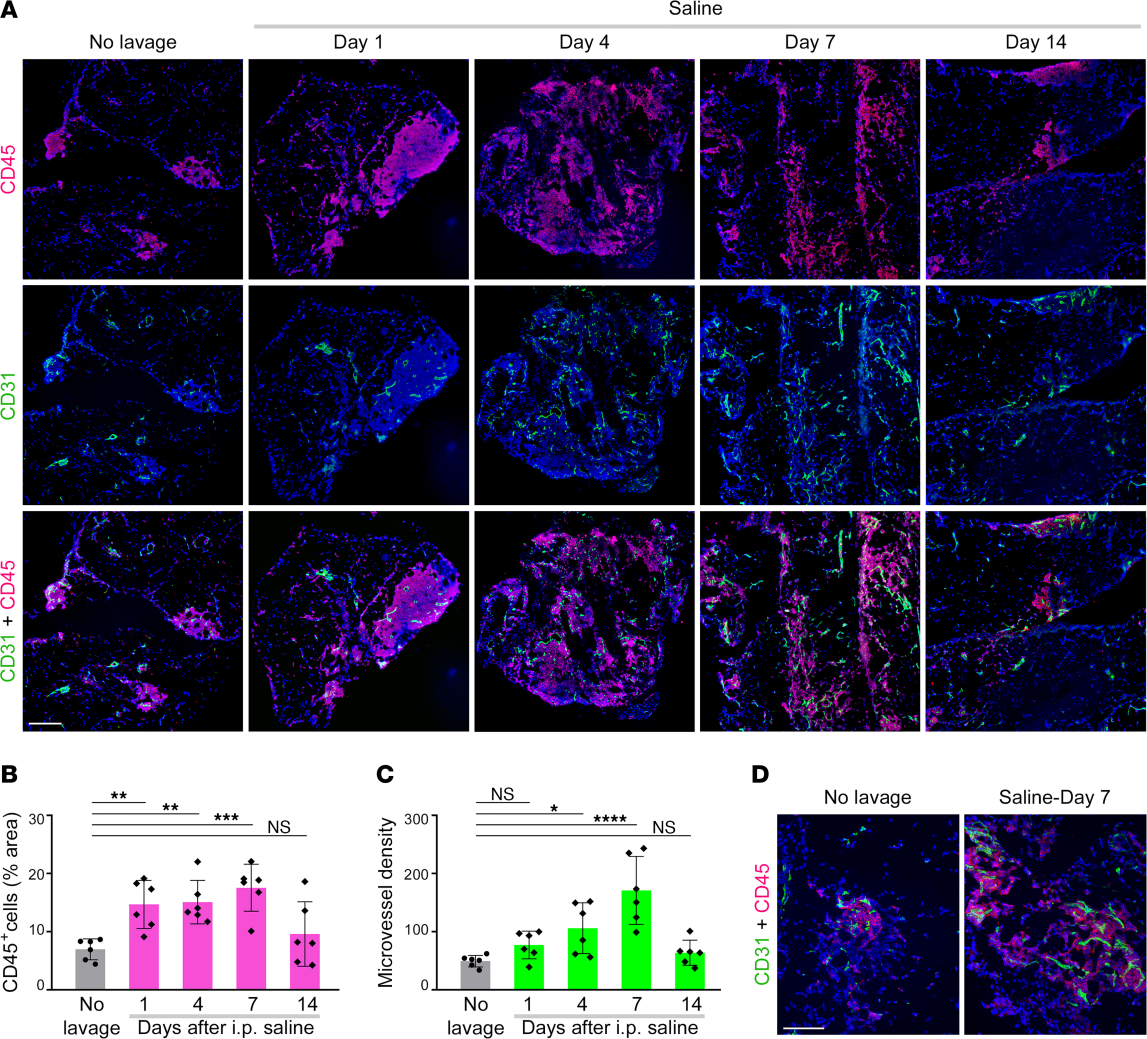
Normal saline stimulates transient expansion of milky spots and neoangiogenesis in the omentum. Immune cells and microvessels were evaluated in omental tissues of untreated mice and mice at 1, 4, 7, and 14 days following i.p. administration of normal saline (*n* = 6 per group). Adult female C57BL/6 mice were used. (**A**) Representative images of CD45 staining (red) and CD31 staining (green) in omental tissues. Scale bar: 200 μm. (**B**) Abundance of immune cells, expressed as the percentage of area of CD45 staining within each tissue section. (**C**) Microvessel density, expressed as the number of CD31^+^ microvessels per 40***×*** microscopic field. In **B** and **C**, an average score for each mouse was calculated by evaluating staining in 4–5 random and independent 40***×*** microscopic fields. (**D**) Higher-magnification images of omental tissues showing prominent localization of microvessels in milky spots. Scale bar: 100 μm. *P < 0.05, **P < 0.01, ***P < 0.001, ****P < 0.0001, by Dunnett’s multiple comparisons test compared with untreated mice (no lavage) in **B** and **C**.

H&E-stained omental tissues of mice showed a striking expansion in milky spots following saline administration (Figure 1C). Quantification of CD45 staining in omental tissues revealed an increase in immune cells at day 1 following saline administration (*P* < 0.01), and this abundance persisted up to day 7, during which time milky spots progressively dispersed (Figure 2, A and B). Increases in CD45^+^ cell counts in the omentum were confirmed by flow cytometry (Supplemental Figure 2D). The number of microvessels, detected by staining the endothelial cell marker CD31, was increased at day 4 following saline administration (*P* < 0.05) and more so at day 7 (*P* < 0.0001) (Figure 2, A and C). Milky spots contain a glomerulus-like network of blood vessels (1). Increased microvessel density was especially prominent in milky spots following saline administration (Figure 2D) and was strongly suggestive of new blood vessel formation. No pronounced increases in lymphatic structures were detected following saline administration (Supplemental Figure 2E). At day 14 following saline administration, immune cell abundance and microvessel density in the omentum had largely returned to baseline levels (Figure 2, A–C).

### Normal saline increases numbers of omental CD11b^int^F4/80^lo^ cells.

**Figure 3:**
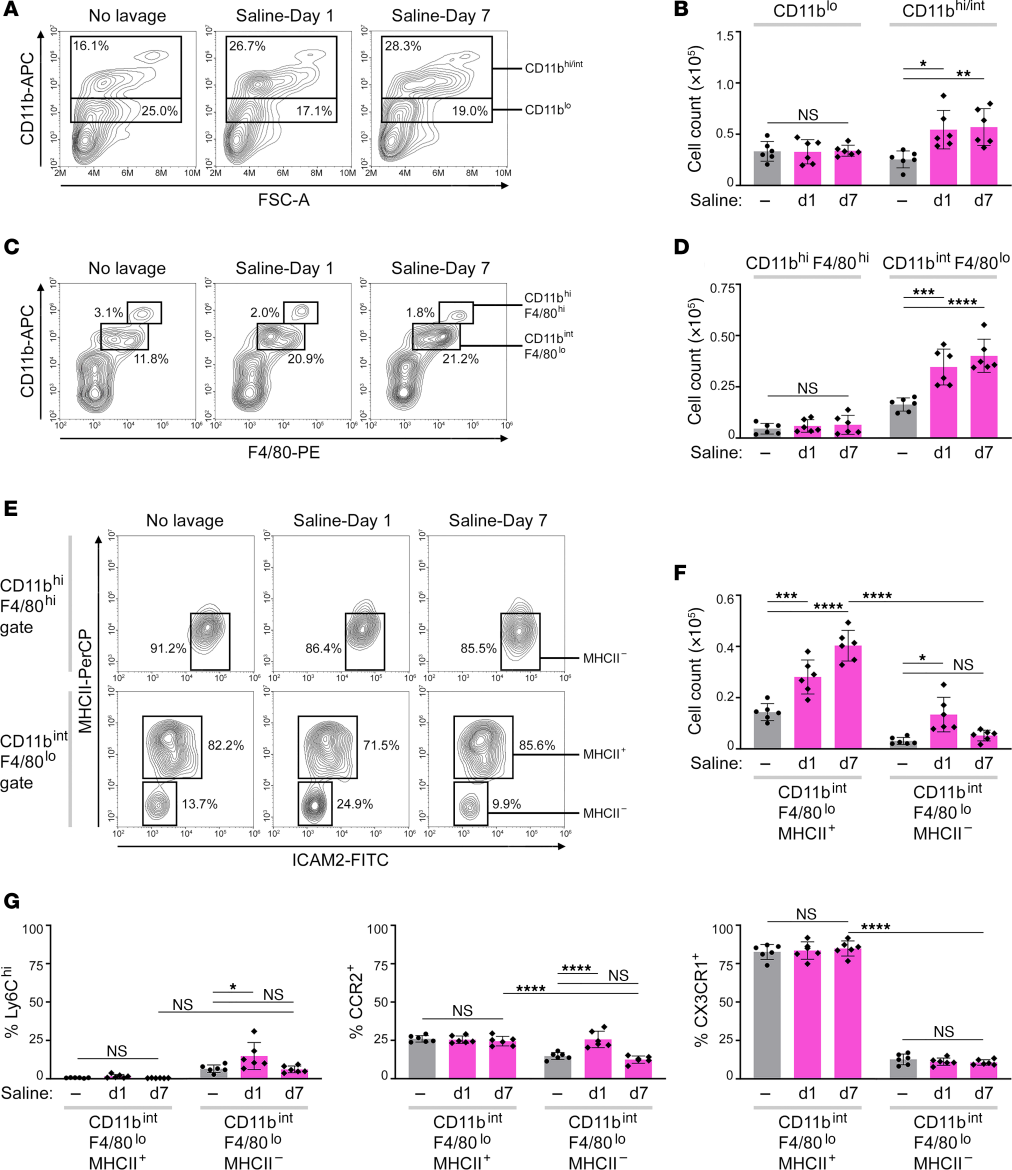
Normal saline increases numbers of omental CD11b^int^F4/80^lo^ SPM-like cells that predominantly express CX3CR1. Flow cytometric analysis of immune cell populations in omental tissues of untreated mice and mice at day 1 and day 7 following i.p. administration of normal saline (*n* = 6 per group). Adult female C57BL/6 mice were used. (**A**) Representative contour plots of forward scatter and CD11b staining within gated CD45^+^ cells, showing abundance of CD11b^lo^ and CD11b^hi/int^ populations. (**B**) Numbers of CD11b^lo^ and CD11b^hi/int^ cells per omental fat band. (**C**) Representative plots of CD11b and F4/80 staining within gated CD45^+^ cells, showing abundance of CD11b^hi^F4/80^hi^ and CD11b^int^F4/80^lo^ populations. (**D**) Numbers of CD11b^hi^F4/80^hi^ and CD11b^int^ F4/80^lo^ cells per omental fat band. (**E**) Representative contour plots of ICAM2 and MHCII staining within gated CD11b^hi^F4/80^hi^ and CD11b^int^F4/80^lo^ populations. The abundance of MHCII^+^ and MHCII^–^ cells within each population is indicated. (**F**) Numbers of CD11b^int^F4/80^lo^ MHCII^+^ and CD11b^int^F4/80^lo^MHCII^–^ cells per omental fat band. (**G**) Percentages of Ly6C^hi^, CCR2^+^, and CX3CR1^+^ cells within gated CD11b^int^F4/80^lo^MHCII^+^ and CD11b^int^F4/80^lo^MHCII^–^ subpopulations. *P < 0.05, **P < 0.01, ***P < 0.001, ****P < 0.0001, by Dunnett’s multiple comparisons test compared with untreated mice for each given cell population in **B** and **D** and by Tukey’s multiple comparisons test in **F** and **G**.

To identify the immune cell population that contributes to the expansion of milky spots following saline administration, we performed immunophenotyping of omental tissues of untreated mice and saline-treated mice at day 1 when milky spot expansion is initially observed, and at day 7 when immune cells and microvessel density are maximally increased. No significant changes were found in the numbers of T and B cells (Supplemental Figure 3A) or cells that express low levels of CD11b (Figure 3, A and B). CD11b^lo^ cells were mostly CD19^+^ (Supplemental Figure 3B), indicating that these are peritoneal B-1 cells (27). By contrast, cells that express high or intermediate levels of CD11b increased following saline administration (*P* < 0.05 at day 1, *P* < 0.01 at day 7) (Figure 3, A and B). Neutrophils constituted only a minor fraction of CD11b^hi/int^ cells and did not increase (Supplemental Figure 3, C and D). No significant changes were observed in the numbers of DCs, NK cells, mast cells, or platelets (Supplemental Figure 3E).

CD11b^hi/int^ cells largely comprised 2 populations that both express CD115 (macrophage CSF receptor) (Supplemental Figure 4A) but express different levels of CD11b and the monocyte/macrophage marker F4/80 (Figure 3C). Following saline administration, CD11b^int^F4/80^lo^ cells but not CD11b^hi^F4/80^hi^ cells significantly increased (*P* < 0.001 at day 1, *P* < 0.0001 at day 7) (Figure 3D). CD11b^hi^F4/80^hi^ and CD11b^int^F4/80^lo^ cells contained lipid-laden vacuoles that are characteristic of adipose tissue macrophages, but the CD11b^int^F4/80^lo^ population comprised macrophages that are smaller in size and also immature cells with monocytic features that were prevalent at day 1 following saline administration (Supplemental Figure 4, B and C). No significant changes in the numbers of CD11b^int^F4/80^lo^ cells or other major types of immune cells were detected in mesenteric and gonadal fat tissues following saline administration (Supplemental Figure 5, A and B).

### Omental CD11b^int^F4/80^lo^ cells predominantly express CX3CR1.

Two broad types of macrophages that reside in the peritoneal cavity have been described. Large peritoneal macrophages (LPM) express high levels of F4/80 and intercellular adhesion molecule 2 (ICAM2) and lack MHCII, whereas small peritoneal macrophages (SPM) express low levels of F4/80 and ICAM2 and are MHCII^+^ (28, 29). Omental CD11b^hi^F4/80^hi^ cells were mostly ICAM2^hi^ and MHCII^–^ (Figure 3E), indicating that these cells are LPM-like. Omental CD11b^int^F4/80^lo^ cells comprised 2 ICAM2^lo^ subpopulations. The most abundant of these subpopulations was MHCII^+^ and SPM-like (Figure 3E). Numbers of CD11b^int^F4/80^lo^MHCII^+^ cells were significantly increased at day 1 (*P* < 0.001) and more so at day 7 (*P* < 0.0001) following saline administration (Figure 3F). CD11b^int^F4/80^lo^MHCII^–^ cells were modestly increased at day 1 (*P* < 0.05) and returned to baseline levels by day 7 (Figure 3F). It is thought that LPM arise from the yolk sac, whereas SPM derive from monocytes (28, 29). Classical inflammatory monocytes express high levels of Ly6C and C-C motif chemokine receptor 2 (CCR2) and low levels of C-X3-C motif chemokine receptor 1 (CX3CR1) (30). The majority of the CD11b^int^F4/80^lo^MHCII^–^ subpopulation lacked CX3CR1, and percentages of Ly6C^hi^ and CCR2^+^ cells increased in this subpopulation at day 1 following saline administration (*P* < 0.05 for Ly6C^hi^, *P* < 0.0001 for CCR2^+^) (Figure 3G and Supplemental Figure 6, A-C). By contrast, the majority of the CD11b^int^F4/80^lo^MHCII^+^ subpopulation lacked Ly6C and CCR2 but strongly expressed CX3CR1, and this antigenic profile was maintained following saline administration (Figure 3G and Supplemental Figure 6, A–C). These findings suggest that the increase in omental CD11b^int^F4/80^lo^ cells following saline administration is initially and only partially due to recruitment of classical monocytes and predominantly stems from the accumulation of CX3CR1^+^ SPM-like cells.

### Normal saline promotes omental neoangiogenesis through increasing CX3CR1^+^ SPM-like cells.

**Figure 4:**
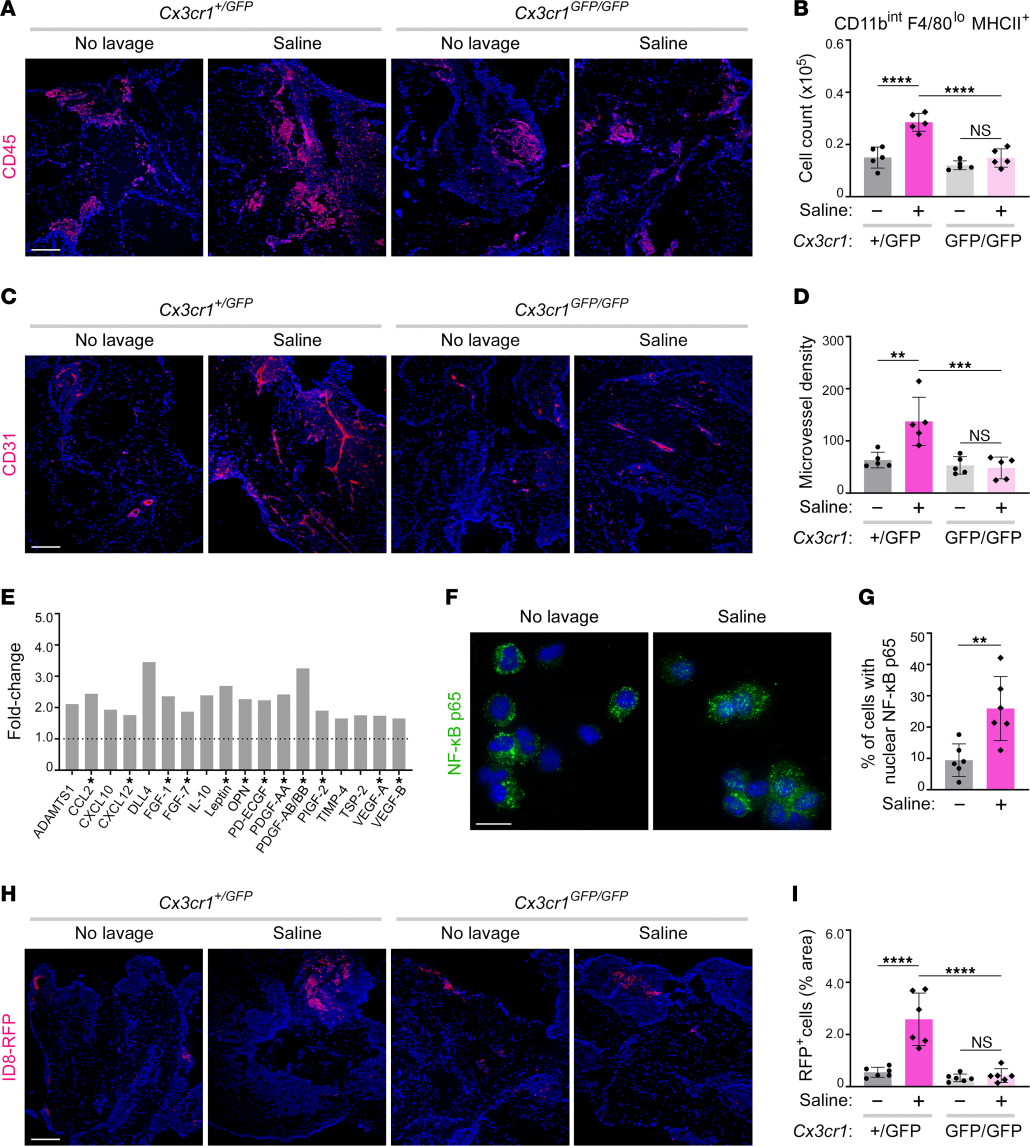
Normal saline promotes omental neoangiogenesis and cancer cell implantation through increasing CX3CR1^+^ SPM-like cells. (**A**–**D**) *Cx3cr1^+/GFP^* and *Cx3cr1^GFP/GFP^* mice were left untreated or administered normal saline (*n* = 5 per group). At 7 days thereafter, omental tissues were evaluated for CD45 staining (**A**), numbers of CD11b^int^F4/80^lo^MHCII^+^ (SPM-like) cells (**B**), CD31 staining (**C**), and microvessel density (**D**). Scale bar in **A** and **C**: 200 μm. (**E**) CX3CR1^+^ SPM-like cells were sorted from omenta of untreated C57BL/6 mice and at day 1 following saline administration (*n* = 5 per group). Lysates of pooled cells of each group were screened on antibody arrays. Shown are proteins with > 1.5-fold higher levels in saline-treated mice relative to untreated mice. Bars represent the averages of 2 replicates. Asterisks indicate proangiogenic proteins. (**F** and **G**) CX3CR1^+^ SPM-like cells were sorted from omenta of untreated C57BL/6 mice and at 1 hour following saline administration (*n* = 6 per group) and were stained for NF-κB p65. (**F**) Representative images of cells. Scale bar: 20 μm. (**G**) Percentages of cells with nuclear NF-κB p65. For each mouse, a minimum of 300 cells were evaluated. (**H** and **I**) *Cx3cr1^+/GFP^* and *Cx3cr1^GFP/GFP^* mice were left untreated or administered saline (*n* = 6 per group). At 7 days thereafter, all groups were inoculated i.p. with ID8 cells that express red fluorescent protein (RFP). Omental colonization was evaluated at 7 days following inoculation. (**H**) Representative images of tissues. Scale bar: 200 μm. (**I**) Tumor foci, expressed as the percentage of area within each tissue. For each mouse, RFP fluorescence was evaluated in 3–5 random and independent 40× microscopic fields. Age-matched adult female mice were used in **A**–**I**. **P < 0.01, ***P < 0.001, ****P < 0.0001, by Tukey’s multiple comparisons test in **B**, **D**, and **I**, by unpaired 2-tailed Student’s *t*-test in **G**.

To evaluate the functional significance of CX3CR1^+^ SPM-like cells, we used *Cx3cr1^GFP^* knockin/knockout (KI/KO) mice in which the *Cx3cr1* gene is disrupted by the insertion of sequences encoding green fluorescent protein (GFP) (31). The vast majority of GFP^+^ cells in the omentum of heterozygotes (*Cx3cr1^+/GFP^*) were SPM-like (CD11b^int^F4/80^lo^MHCII^+^), and this was consistent with the predominant expression of CX3CR1 in CD11b^int^F4/80^lo^MHCII^+^ cells in the omentum of C57BL/6 mice (Supplemental Figure 7, A and B). Following saline administration, milky spots expanded in *Cx3cr1^+/GFP^* mice but not in homozygotes (*Cx3cr1^GFP/GFP^*) that are devoid of functional CX3CR1 (Figure 4A). Numbers of CD11b^int^F4/80^lo^MHCII^+^ cells were significantly increased in the omentum of *Cx3cr1^+/GFP^* mice (*P* < 0.0001) but not in *Cx3cr1^GFP/GFP^* mice following saline administration (Figure 4B). No significant differences were observed in cell counts in other major immune cell populations in the omentum of *Cx3cr1^+/GFP^* and *Cx3cr1^GFP/GFP^* mice following saline administration (Supplemental Figure 7C).

Following saline administration, microvessel density significantly increased in the omentum of *Cx3cr1^+/GFP^* mice (*P* < 0.01) but not in *Cx3cr1^GFP/GFP^* mice (Figure 4, C and D). These findings implied that CX3CR1^+^ SPM-like cells have proangiogenic properties. To gain further insight, CX3CR1^+^ SPM-like cells were sorted from omental tissues of untreated and saline-treated mice (Supplemental Figure 7D), and lysates of equivalent numbers of pooled cells from each group were screened for angiogenesis-associated proteins by using an antibody array. Forty-two of the 53 proteins represented on the array were detected in CX3CR1^+^ SPM-like cells of untreated mice (Supplemental Figure 7E). Levels of 18 proteins were increased by > 1.5-fold in CX3CR1^+^ SPM-like cells of saline-treated mice (Figure 4E). Of these 18 proteins, some are antiangiogenic such as Delta-like protein 4 (DLL4) and thrombospondin-2 (TSP-2), but 12 have well-characterized proangiogenic properties. These include C-C motif chemokine ligand 2 (CCL2), platelet-derived growth factor A (PDGF-A), PDGF-B, fibroblast growth factor (FGF)-1, VEGF-A, VEGF-B, and osteopontin (OPN) (Figure 4E). Increased nuclear localization of NF-κB p65, which regulates a number of angiogenesis-associated genes (32–35), was detected in CX3CR1^+^ SPM-like cells of saline-treated mice (Figure 4, F and G). These findings indicate that saline administration stimulates neoangiogenesis in the omentum by promoting accumulation of CX3CR1^+^ SPM-like cells that constitutively express a repertoire of proangiogenic factors and by increasing expression of a subset of these factors in CX3CR1^+^ SPM-like cells.

### Saline-stimulated accumulation of CX3CR1^+^ SPM-like cells promotes implantation of cancer cells onto the omentum.

Angiogenesis promotes tumor growth, and cancer cells that circulate in the peritoneal fluid are prone to implant in milky spots (6, 7). In view of our findings that saline administration stimulates the accumulation of CX3CR1^+^ SPM-like cells in the omentum and that these cells promote neoangiogenesis, we investigated whether saline administration promotes implantation of cancer cells onto the omentum through increasing CX3CR1^+^ SPM-like cells. Groups of adult female *Cx3cr1^+/GFP^* and *Cx3cr1^GFP/GFP^* mice were either administered normal saline or left untreated and, at 7 days thereafter, were inoculated i.p. with ID8 mouse ovarian cancer cells. At 7 days following cancer cell inoculation, microscopic foci were detected in the omentum of untreated groups of *Cx3cr1^+/GFP^* and *Cx3cr1^GFP/GFP^* mice (Figure 4, H and I). A significant increase in tumor foci in the omentum was observed in saline-treated *Cx3cr1^+/GFP^* mice (*P* < 0.0001) but not in saline-treated *Cx3cr1^GFP/GFP^* mice (Figure 4, H and I). These findings implicate that normal saline renders the omentum conducive for implantation of cancer cells by increasing the accumulation of proangiogenic CX3CR1^+^ SPM-like cells.

### Normal saline increases expression of the CX3CR1 ligand within and surrounding the omental vasculature.

**Figure 5:**
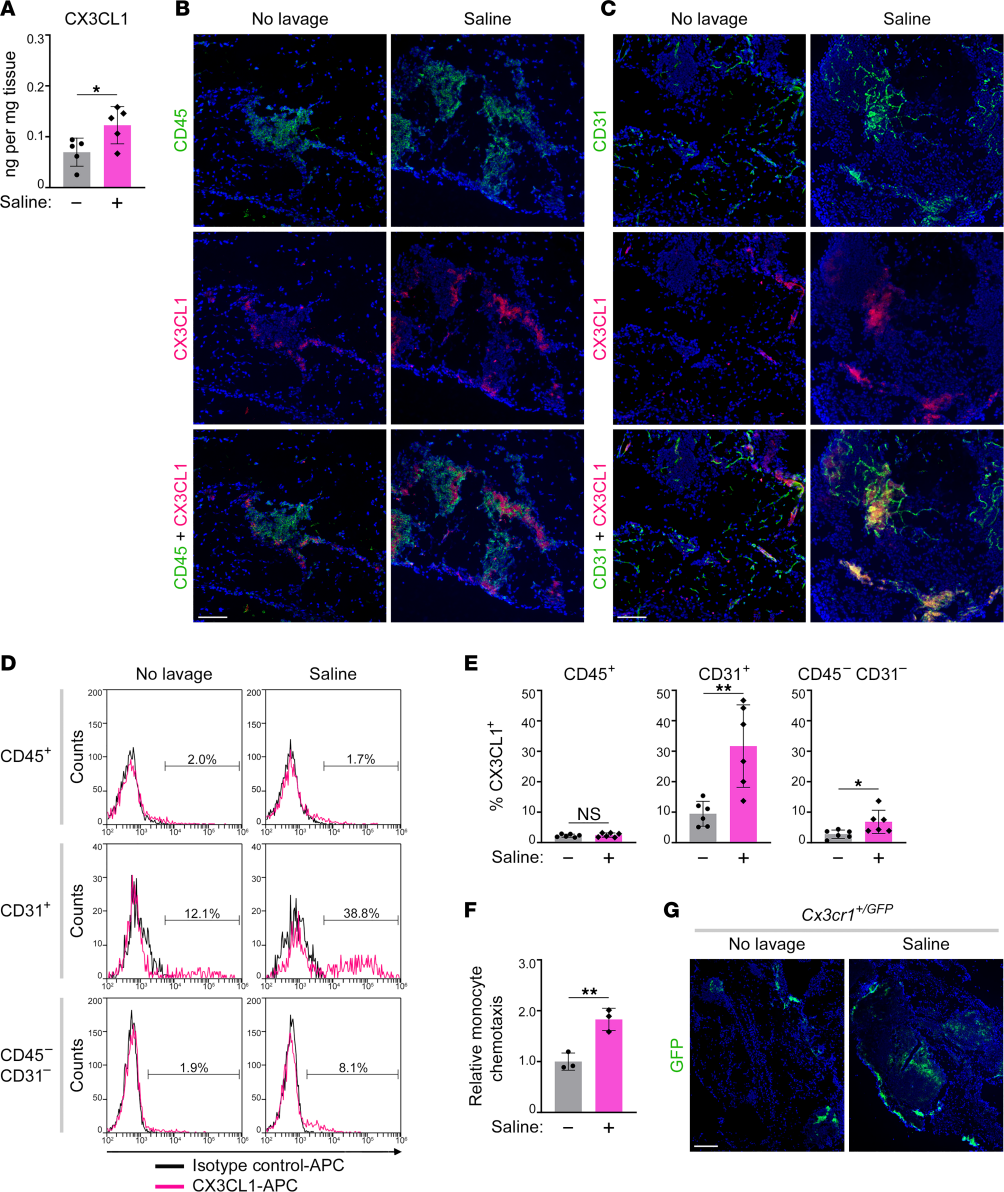
Normal saline increases expression of CX3CL1 within and surrounding the omental vasculature. (**A**–**E**) CX3CL1 expression in omental tissues of untreated mice and at day 1 following saline administration. Adult female C57BL/6 mice were used. (**A**) Total CX3CL1 content in omental tissues, determined by ELISA. Data of *n* = 5 mice per group are shown. (**B** and **C**) Representative images of immunofluorescence staining of CD45 and CX3CL1 (**B**), and CD31 and CX3CL1 (**C**). Scale bar: 100 μm. (**D**) Representative histogram plots of staining of membrane-bound CX3CL1 within gated CD45^+^, CD31^+^, and CD45^–^CD31^–^ populations. (**E**) Percentages of CX3CL1^+^ cells within the indicated gated populations. Data of *n* = 6 mice per group are shown. (**F**) GFP^+^ blood monocytes of *Cx3cr1^+/GFP^* mice were assayed for chemotaxis toward equivalent numbers of CD45^–^ cells of the stromal vascular fraction that were sorted from omenta of C57BL/6 mice left untreated and at day 1 following saline administration. Shown are data of 3 independent assays, where each assay used omental stromal vascular cells from a different mouse. (**G**) Representative images of omental tissues of adult female *Cx3cr1^+/GFP^* mice that were left untreated and at day 7 following saline administration. Scale bar: 200 μm. Tissues of *n* = 5 mice per group were evaluated. *P < 0.05, **P < 0.01, by unpaired 2-tailed Student’s *t* test in **A**, **E** and **F**.

C-X3-C motif chemokine ligand 1 (CX3CL1), the sole ligand of CX3CR1, is predominantly expressed as a membrane-bound protein in endothelial cells (36) and also in smooth muscle cells (37). Because the prominent response in the omentum to saline administration is an accumulation of CX3CR1^+^ SPM-like cells, we investigated whether saline induces an early increase in local CX3CL1 levels. The CX3CL1 content in the omentum increased 2-fold (*P* < 0.05) at day 1 following saline administration (Figure 5A). CX3CL1 was detected in milky spots and focally near the submesothelial layer and mostly though not exclusively colocalized with microvessels (Figure 5, B and C). These findings were confirmed by flow cytometric analysis of membrane-associated CX3CL1. Membrane-bound CX3CL1 was almost undetectable in immune cells but was markedly increased in endothelial cells (*P* < 0.01) and more modestly in nonendothelial stromal cells (*P* < 0.05) following saline administration (Figure 5, D and E). To evaluate the significance of CX3CL1 induction on chemotaxis, we used *Cx3cr1^+/GFP^* mice that have normal blood monocyte counts and have been used for tracing CX3CR1^+^ monocytes (31, 38, 39). GFP^+^ blood monocytes of *Cx3cr1^+/GFP^* mice were assayed for chemotaxis toward CD45^–^ cells of the stromal vascular fraction of omental tissues of untreated and saline-treated C57BL/6 mice. Monocytes showed greater chemotaxis toward omental stromal vascular cells of saline-treated mice than of untreated mice in vitro (*P* < 0.01) (Figure 5F) and were increased in the omentum of *Cx3cr1^+/GFP^* mice following saline administration in vivo (Figure 5G). These findings suggest that saline administration stimulates the recruitment of CX3CR1^+^ monocytes to the omentum, at least in part, by inducing CX3CL1 expression within and surrounding the omental vasculature.

### LRS is less deleterious to mesothelial integrity than normal saline.

**Figure 6:**
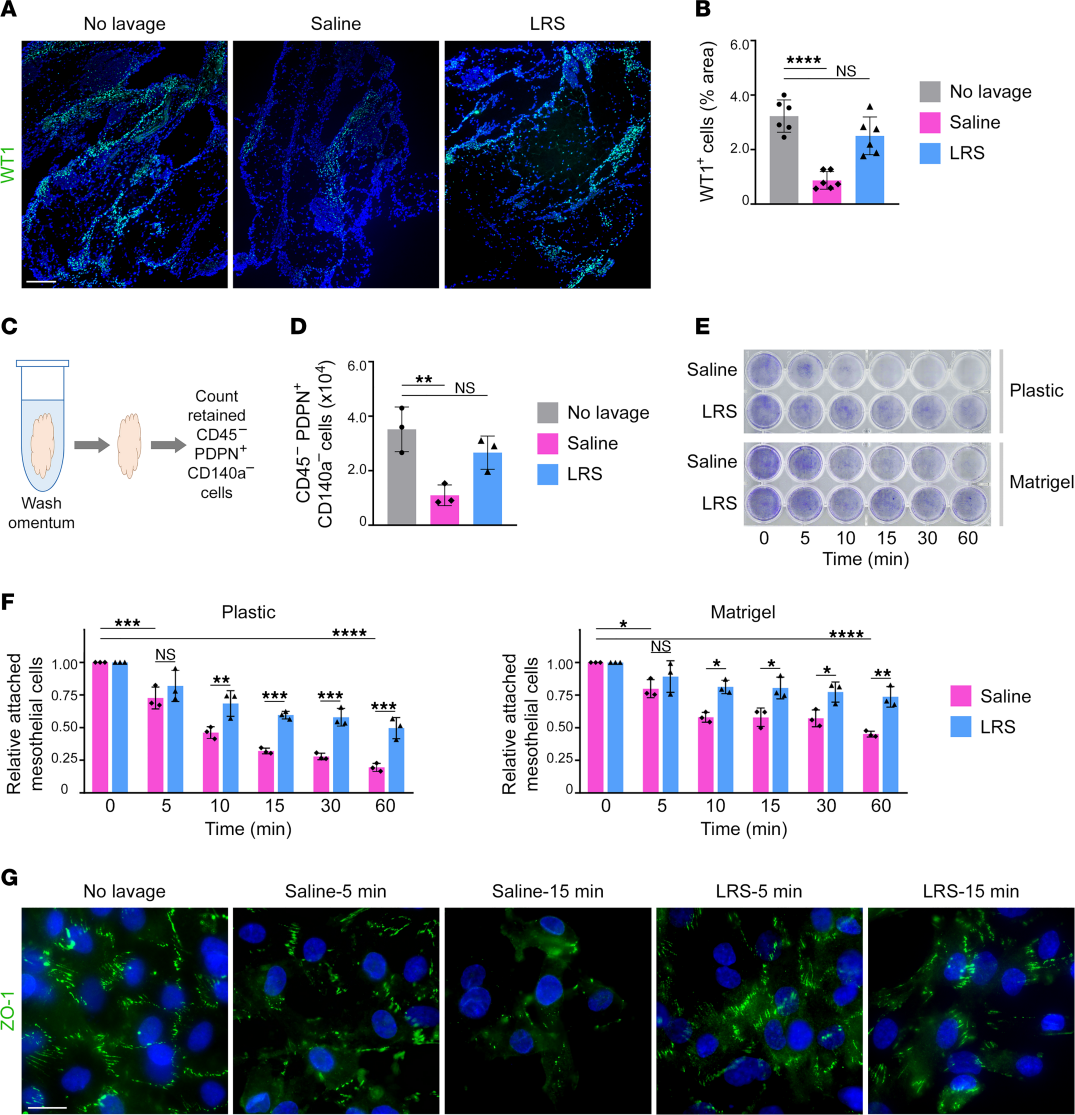
LRS is less deleterious to mesothelial integrity than normal saline. (**A** and **B**) In vivo analysis of mesothelial cell exfoliation. WT1^+^ cells were evaluated in omental tissues of untreated mice and at day 1 following i.p. administration of normal saline or LRS at the same dosage (12.5 mL/kg) (*n* = 6 per group). Adult female C57BL/6 mice were used. (**A**) Representative images of WT1 staining. Scale bar: 200 μm. (**B**) Abundance of WT1^+^ cells, expressed as the percentage of area within omental tissue. Data of untreated and saline-treated groups is duplicated in Figure 1E. (**C** and **D**) Ex vivo analysis of mesothelial cell exfoliation. (**C**) Whole naive omental tissues of adult female C57BL/6 mice were excised and incubated in 2.0 mL of saline or LRS for 1 hour with shaking and in parallel or were left untreated (*n* = 3 per group). Mesothelial cells that were retained in tissues were quantified by flow cytometric analysis of the CD45^–^PDPN^+^CD140a^–^ population (Supplemental Figure 2B). (**D**) Numbers of retained CD45^–^PDPN^+^CD140a^–^ cells per omental fat band. (**E**–**G**) In vitro analysis of mesothelial cell exfoliation. Human omental mesothelial cells were plated in plastic or Matrigel-coated 24-well plates and then incubated in 0.5 mL of saline or LRS for the indicated times. Cells that remained attached were detected by crystal violet staining (**E**) and quantified by measuring absorbance (**F**). Data of 3 independent experiments is shown in **F**. (**G**) ZO-1 staining in untreated and treated cells cultured on plastic chamber slides. Scale bar: 20 μm. Shown are representative images of 3 independent experiments. *P < 0.05, **P < 0.01, ***P < 0.001, ****P < 0.0001, by Dunnett’s multiple comparisons test compared with no lavage in **B** and **D** and by Tukey’s multiple comparisons test in **F**.

In addition to its use in lavage, normal saline is commonly used for fluid resuscitation. Several clinical trials have shown that i.v. infusion using balanced crystalloids such as LRS causes fewer adverse effects than normal saline (21, 40–42). Comparison of the chemical composition of LRS with that of normal saline and body fluids is shown in Supplemental Table 1. We investigated whether LRS induces the same responses in the omentum as normal saline when administered i.p. as a lavage solution to C57BL/6 mice. In contrast to normal saline, LRS did not decrease peritoneal pH (Supplemental Figure 8A). No significant differences in lactate levels in the peritoneal fluid and peripheral blood were detected at day 1 following administration of LRS (Supplemental Figure 8B). Whereas omental surfaces were denuded of mesothelial cells at day 1 following administration of saline, the mesothelium was mostly preserved following administration of LRS (Figure 6, A and B). To confirm these findings, whole naive omental tissues were washed in normal saline or LRS ex vivo. Thereafter, viable mesothelial cells that were retained in tissues were quantified (Figure 6C). As compared with the unwashed omentum, numbers of mesothelial cells in the omentum were significantly decreased by washing with saline (*P* < 0.01) but not with LRS (Figure 6D). To confirm our findings in human cells, primary cultures of human omental mesothelial cells were treated with either saline or LRS. Saline rapidly stripped mesothelial cells from culture plates, whereas less stripping was observed with LRS (Figure 6, E and F). Because mesothelial cells overlie a basement membrane (8), we repeated the assay by using mesothelial cells that were plated on a basement membrane matrix and obtained similar results (Figure 6, E and F). The greater loss of cell adhesion in saline-treated cells, as compared with LRS-treated cells, was confirmed by the downregulation of the tight junction protein ZO-1 (Figure 6G). These findings demonstrate that LRS causes substantially less mesothelial cell exfoliation than normal saline.

### LRS does not stimulate accumulation of CX3CR1^+^ SPM-like cells, omental neoangiogenesis or implantation of cancer cells.

**Figure 7:**
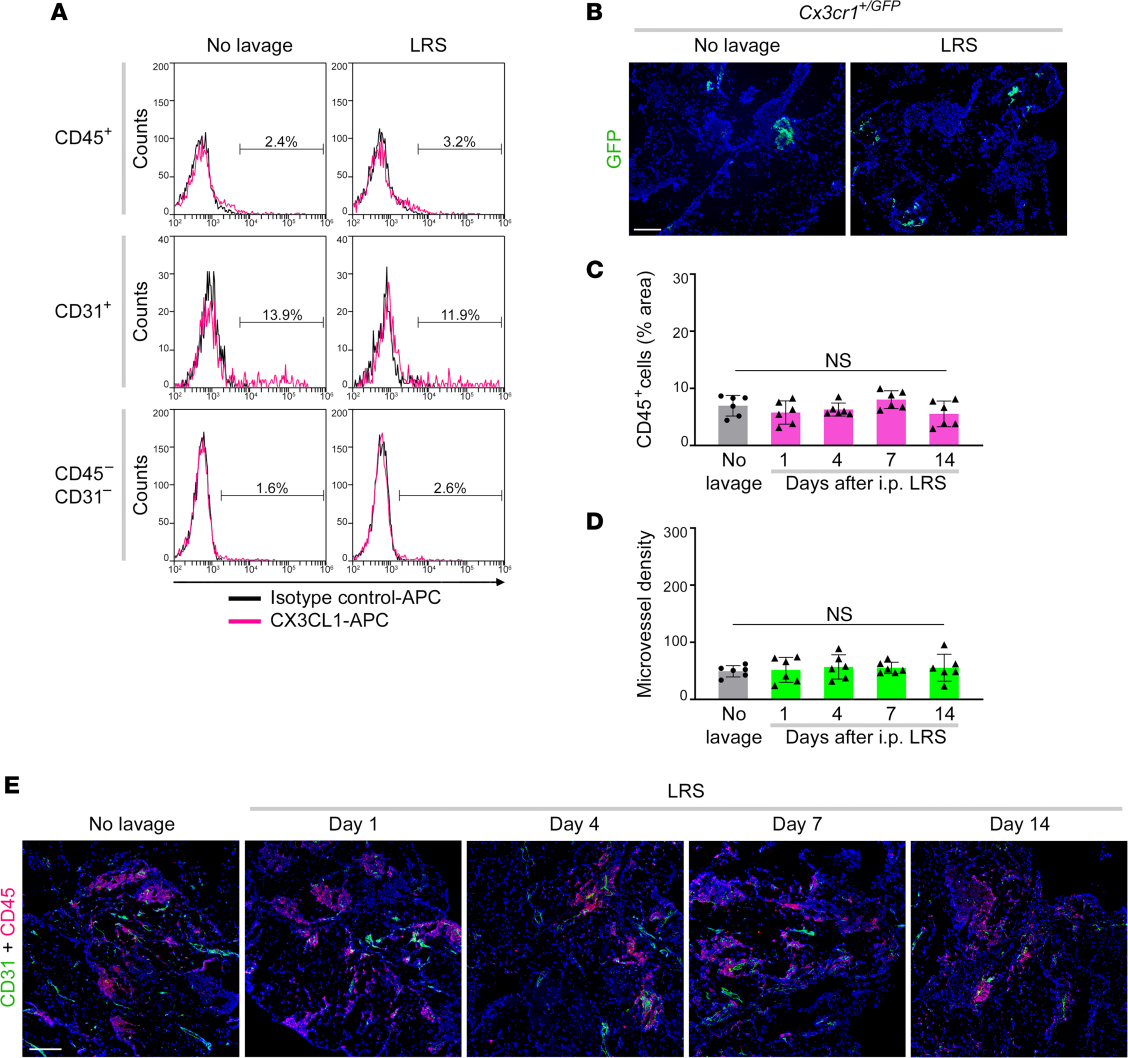
LRS does not stimulate CX3CL1 expression, accumulation of CX3CR1^+^ SPM-like cells, or omental neoangiogenesis. (**A**) Representative histogram plots of staining of membrane-bound CX3CL1 within gated CD45^+^, CD31^+^, and CD45^–^CD31^–^ populations in omental tissues of adult female C57BL/6 mice that were left untreated and at day 1 following i.p. administration of LRS. Tissues of *n* = 6 mice per group were evaluated. (**B**) Representative images of omental tissues of adult female *Cx3cr1^+/GFP^* mice that were left untreated and at day 7 following LRS administration. Scale bar: 200 μm. Tissues of *n* = 5 mice per group were evaluated. (**C**–**E**) Abundance of immune cells (**C**) and microvessels (**D**) were evaluated in omental tissues of adult female C57BL/6 mice that were left untreated and at 1, 4, 7, and 14 days following i.p. administration of LRS (*n* = 6 per group) as described in Figure 2. Data of the untreated group are duplicated in Figure 2, B and C. (**E**) Representative images of CD45 and CD31 staining in omental tissues. Scale bar: 200 μm. Data in **C** and **D** were evaluated by Dunnett’s multiple comparisons test compared with untreated mice (no lavage).

We next evaluated the impact of i.p. administration of LRS on other cell populations in the omentum. In contrast to normal saline, LRS did not stimulate CX3CL1 expression in endothelial cells or other stromal cells of the omentum (Figure 7A). Consistent with this finding, CX3CR1^+^ cells did not accumulate in the omentum following LRS administration (Figure 7B). LRS administration did not significantly change immune cell abundance in the omentum (Figure 7C) or cell counts in immune cell populations in the omentum and other peritoneal fat depots (Supplemental Figure 9, A–C). Furthermore, LRS administration did not significantly change microvessel density in the omentum (Figure 7, D and E). No other histologic changes were observed in the omentum or other peritoneal fat depots of LRS-treated mice (Supplemental Figure 9D).

**Figure 8:**
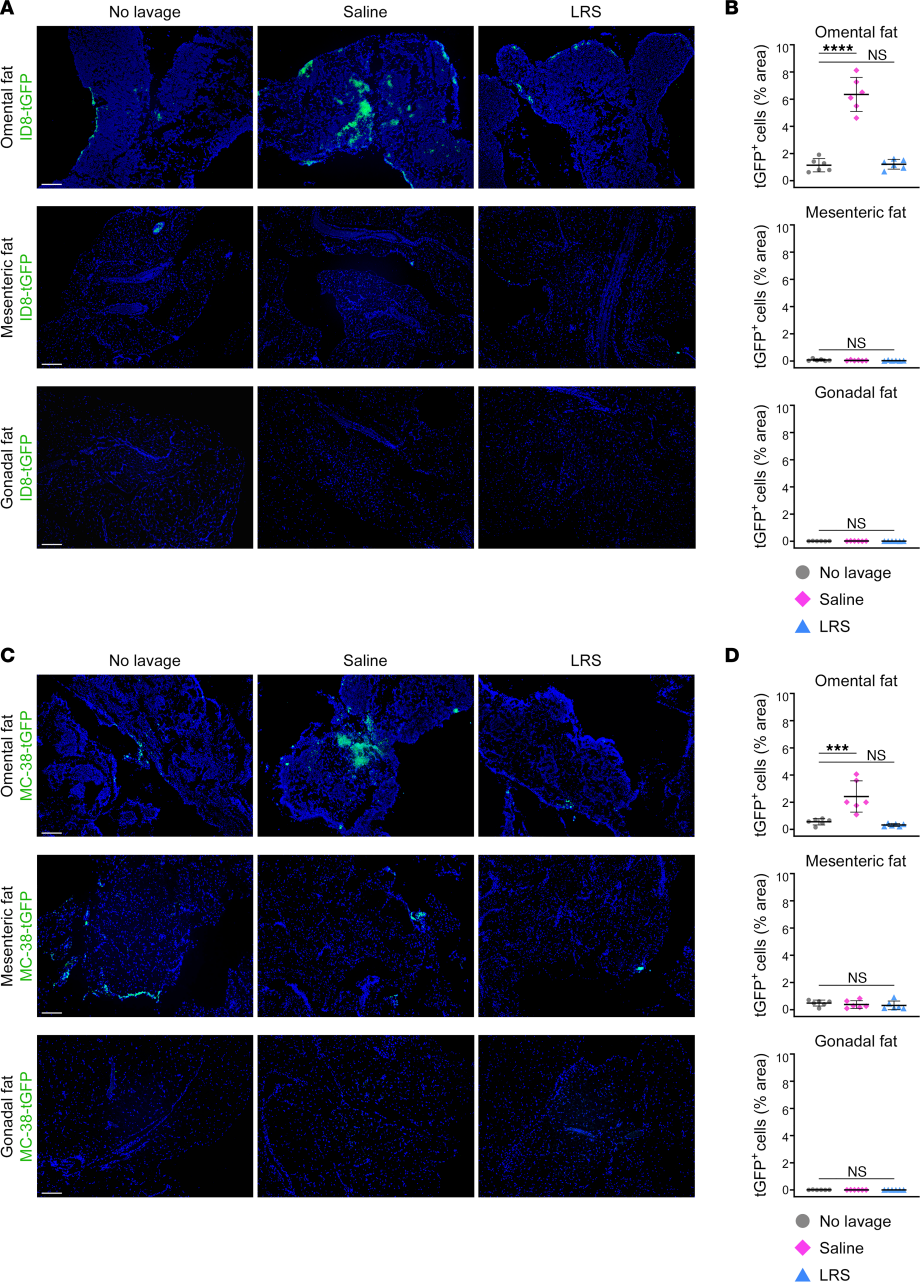
LRS does not stimulate implantation of cancer cells onto the omentum. Age-matched adult female C57BL/6 mice were randomized into 3 groups and administered either normal saline or LRS i.p. at the same dosage (12.5 mL/kg) or were left untreated (*n* = 6 per group). At 7 days thereafter, all groups were inoculated i.p. with mouse cancer cells that express turbo GFP (tGFP). (**A** and **B**) Implantation of ID8-tGFP ovarian cancer cells on omental, mesenteric, and gonadal fat tissues at 7 days following cancer cell inoculation. (**A**) Representative images of tissues. Scale bar: 200 μm. (**B**) Abundance of ID8-tGFP cells, expressed as the percentage of area within each tissue. An average score for each tissue of each mouse was calculated by evaluating tGFP fluorescence in 3–5 random and independent 40× microscopic fields. (**C** and **D**) Implantation of MC-38-tGFP colon adenocarcinoma cells on omental, mesenteric, and gonadal fat tissues at 2 days following cancer cell inoculation. (**C**) Representative images of tissues. Scale bar: 200 μm. (**D**) Abundance of MC-38-tGFP cells, evaluated as in **B**. ***P < 0.001, ****P < 0.0001, by Dunnett’s multiple comparisons test compared with untreated mice (no lavage) in **B** and **D**.

We subsequently evaluated whether LRS has a less deleterious effect on cancer cell implantation than normal saline. Adult female C57BL/6 mice were randomized into groups and were either administered saline or LRS or left untreated. Seven days afterward, all groups were inoculated i.p. with ID8 mouse ovarian cancer cells. At 7 days following cancer cell inoculation, microscopic foci were detected on the omentum of untreated mice (Figure 8, A and B). Saline-treated mice showed a 6-fold increase in colonization (*P* < 0.0001) and prominent intraomental foci (Figure 8, A and B). By contrast, colonization was not increased in LRS-treated mice, and microscopic foci were confined to the omental surface (Figure 8, A and B). Colonization of the mesentery and gonadal fat tissues was minimal and was not significantly affected by either saline or LRS (Figure 8, A and B). Similar results were obtained when groups of untreated, saline-treated, and LRS-treated mice were inoculated i.p. with MC-38 mouse colon adenocarcinoma cells (Figure 8, C and D). These findings demonstrate that normal saline renders the omentum conducive for the implantation of cancer cells, whereas LRS does not.

## Discussion

The benefit of omentectomy in patients with ovarian cancer who present without overt metastasis has been questioned (10, 15, 16), and the omentum is often partially preserved in patients with gastric cancer (11, 17, 43). However, cancer cells that circulate in the peritoneal fluid have a predilection for implanting in milky spots, and there are no effective strategies to minimize the risk of occult cancer cells colonizing omental tissues that are preserved. Normal saline is widely used for diagnostic and intraoperative lavage of the peritoneal cavity. The ability of the omentum to absorb peritoneal fluid was discovered > 100 years ago (2), but its significance has been greatly underappreciated. Here, we report that normal saline, when administered into the peritoneal cavity of mice, is prominently absorbed by the omentum and increases the receptivity of the omentum for cancer cells. Importantly, our study shows that the risk of omental colonization is minimized by using a more biocompatible crystalloid instead of normal saline as a lavage solution.

Our study implicates a model in which normal saline “primes” the omentum for colonization through a sequence of remodeling events that is initiated by mesothelial cell exfoliation. Mesothelial cells line serous cavities and internal organs, and exfoliated mesothelial cells are often detected in peritoneal washings (9, 20). Our findings suggest that the omental mesothelium is highly susceptible to exfoliation by normal saline because of the immense capability of the omentum to absorb fluid. By comparison, the mesentery and gonadal fat pads showed weaker absorptive ability and minimal mesothelial cell exfoliation. Lavage procedures can mechanically exfoliate mesothelial cells (20), but shear stress does not fully explain the removal of mesothelial cells because the administration of LRS causes minimal exfoliation. The sodium content of normal saline is slightly higher (i.e., ~10% higher) than that of serum and interstitial fluids, but its chloride content is nearly 50% above physiological levels (21, 42, 44–47) (Supplemental Table 1). When used for infusion, normal saline can cause adverse renal and vascular effects that are associated with hyperchloremic acidosis (21, 40–42). By contrast, LRS is buffered and its sodium and chloride content is within the physiological range (21, 42) (Supplemental Table 1). Several clinical trials in the critical care setting have reported that adverse effects associated with hyperchloremic acidosis are minimized when balanced crystalloids such as LRS are used instead of normal saline for infusion (21, 40–42). It is possible that the deleterious effect of normal saline on mesothelial cell adhesion stems from hyperchloremia and that the biocompatibility of LRS minimizes exfoliation of these cells.

Little is known about the impact of normal saline on underlying visceral tissues. The present study shows that normal saline stimulates a transient expansion in milky spots, and this expansion is largely due to increased numbers of CD11b^int^F4/80^lo^cells that are CX3CR1^+^ and have hallmarks of monocyte-derived SPM. Whereas classical Ly6C^hi^CCR2^+^ monocytes are rapidly recruited to sites of inflammation, CX3CR1^+^ monocytes patrol the vascular lumen (30). Our findings implicate that normal saline increases SPM-like cells in the omentum, at least in part, by stimulating the recruitment of blood monocytes through inducing CX3CL1 expression within and surrounding the omental vasculature. These monocytes likely differentiate into adipose tissue–associated SPM-like cells, as most omental CD11b^int^F4/80^lo^ cells at day 7 following saline administration contain lipid-laden vacuoles. We have found that ~80% of SPM in the peritoneal fluid lack CX3CR1 (H. Akasaka & H. Naora, unpublished observations) but cannot exclude the possibility that some of these circulating SPM mobilize to the omentum following saline administration. Although the vast majority of CX3CR1^+^ cells in the omentum were found to be SPM-like, other types of cells might also be recruited. DCs, NK cells, mast cells, and platelets respond to CX3CL1 (48) but constitute minor cell populations in the omentum, and their counts did not significantly increase following saline administration. Some T cells also respond to CX3CL1 (48), but T cell counts did not increase in saline-treated mice. The mechanism by which saline stimulates CX3CL1 expression is unclear but might be related to coagulation. It has been reported that saline stimulates mesothelial cells to release tissue factor, the initiator of coagulation (24), and that thrombin induces CX3CL1 expression in endothelial cells (49).

CX3CR1^+^ monocytes/macrophages have been shown to mediate wound healing in various types of tissues (39, 50). It is likely that CX3CR1^+^ SPM-like cells mediate tissue repair in the omentum following saline administration, as these cells accumulated following mesothelial cell exfoliation, persisted until the mesothelium had regenerated, and were detected near the submesothelial layer as well as in milky spots. CX3CR1^+^ SPM-like cells were found to constitutively express a wide repertoire of proangiogenic cytokines and proteases, and local levels of these proteins could conceivably increase as SPM-like cells accumulate in the omentum. Our findings also suggest that local levels of several proangiogenic factors such as CCL2, PDGF-B, VEGF-A, and OPN could be further increased through saline-stimulated production of these factors by SPM-like cells. Genes that encode CCL2, PDGF-B, VEGF-A, and OPN are transcriptionally activated by NF-κB (32–35). NF-κB activity depends on its translocation to the nucleus, and increased nuclear localization of NF-κB p65 was detected in SPM-like cells following saline administration. Normal saline has a low pH, and it has been reported that low pH stimulates NF-κB activity in macrophages (51). Collectively, these findings suggest that CX3CR1^+^ SPM-like cells mediate tissue repair in the omentum following saline-induced mesothelial cell exfoliation by providing a repertoire of proangiogenic factors, of which a subset are elevated through saline-stimulated NF-κB activation. Although other types of immune cells did not significantly increase in number following saline administration, the possibility cannot be excluded that saline might also stimulate NF-κB activity and expression of proangiogenic factors in these cells.

A discovery in the present study is that the administration of normal saline into the peritoneal cavity stimulates colonization of the omentum by cancer cells that circulate in the peritoneal fluid and that this colonization is facilitated, at least in part, by the proangiogenic properties of CX3CR1^+^ SPM-like cells. By contrast, the administration of LRS did not increase CX3CR1^+^ SPM-like cells or promote neoangiogenesis, and it did not stimulate colonization of the omentum. LRS might also limit colonization by decreasing inflammation. A clinical trial found that C-reactive protein levels were lower in patients with acute pancreatitis following resuscitation with LRS than with normal saline (52). LRS contains lactate that is metabolized to bicarbonate, the key buffering component in body fluids (21, 42), and a caveat is that elevated lactate levels might fuel growth of implants. One trial reported a modest increase (~4%) in serum lactate levels in healthy subjects who received i.v. LRS (53), but no significant increase in lactate levels was reported in another trial (54). In the present study, LRS did not stimulate tumor implant growth, and lactate levels were not significantly increased in peripheral blood and peritoneal fluid of mice that received i.p. LRS.

In summary, the present study highlights the need to carefully consider the absorptive ability of the omentum and the effect of lavage solutions in cancer patients whose omental tissues are preserved. Our findings raise the possibility that the use of normal saline as a lavage solution in the peritoneal cavity increases the risk of omental colonization by occult cancer cells that circulate in the peritoneal fluid and that this risk can be minimized by alternatively using a biocompatible crystalloid for lavage procedures. Biocompatible lavage solutions might also be beneficial for cytological evaluation of peritoneal washings by minimizing the content of mesothelial cells.

## Methods

### Reagents.

Normal saline was prepared by dissolving sodium chloride (Sigma-Aldrich) at a final concentration of 0.9% in double deionized water, followed by filtration through a 0.2 μm filter and autoclaving. Sterile LRS was purchased from Dechra Veterinary Products. Prior to use, samples of each solution were tested for pH. Antibodies are described in Supplemental Table 2. Sources of other reagents were as follows: Fixable Viability Dye eFluor 660 dye (Thermo Fisher Scientific); DAPI, Evans Blue dye, H&E solution, and Giemsa solution (Sigma-Aldrich); and collagenase type D, deoxyribonuclease I (DNase I) (Worthington Biochemical Corp.).

### Mouse cell lines.

The parental ID8 cell line was obtained from Katherine Roby (University of Kansas Medical Center, Kansas City, Kansas, USA). ID8 cells that stably express RFP or turboGFP (tGFP) have been previously described (7). The parental MC-38 cell line was obtained from Ronald DePinho (University of Texas MD Anderson Cancer Center) with permission from Jeffrey Schlom (National Cancer Institute). ID8 and MC-38 cells were cultured in DMEM supplemented with 10% FBS, 100 units/mL penicillin, and 100 μg/mL streptomycin (GenDEPOT). To generate MC-38 cells that stably express tGFP, MC-38 cells were transfected with pGFP-V-RS vector (Origene) by using Lipofectamine 3000 reagent (Invitrogen), followed by selection with 2 μg/mL puromycin (Sigma-Aldrich).

### Human mesothelial cells.

Mesothelial cells that were isolated from normal omental tissues of women who underwent surgery for benign conditions have been previously described (55). Mesothelial cells were cultured in RPMI 1640 medium supplemented with 20% FBS, 100 units/mL penicillin, 100μg/mL streptomycin (GenDEPOT), 0.5 mM sodium pyruvate, 1× minimum essential medium (MEM) nonessential amino acids, 1× MEM vitamins (Sigma-Aldrich).

### Animals.

C57BL/6 mice (stock no. 000664) and a breeder pair of *Cx3cr1^GFP/GFP^* mice (stock no. 005582, B6.129P2[Cg]-*Cx3cr1^tm1Litt/^*J) were purchased from The Jackson Laboratory and housed under barrier conditions. *Cx3cr1^GFP/GFP^* mice have been previously described (31). In these mice, the first 390 bp of exon 2 of the *Cx3cr1* gene is replaced with sequences encoding enhanced GFP. Eight- to 10-week-old female mice were used in all experiments.

### Administration of lavage solutions.

Prior to injection, each mouse was weighed and the abdomen was sterilized by an alcohol swab. Mice were randomized into groups and were either administered a single dose of normal saline or LRS at 12.5 mL/kg body weight by i.p. injection using a 30-gauge needle or left untreated. Mice were euthanized at time points indicated in the text. Where pH and lactate levels were measured, mice were euthanized by cervical dislocation to avoid CO_2_ acidosis. In all other experiments, mice were euthanized by CO_2_ asphyxiation.

### Measurement of pH.

pH in the peritoneal cavity of mice was measured by using a Premium-Series PH60S meter (Apera Instruments). The spear probe was placed in 5 predetermined sites in the cavity to take pH measurements (Supplemental Figure 1B). These 5 measurements were used to calculate an average pH for each mouse. Two-point calibration (pH 7.0 and 4.0) of the instrument was performed prior to use.

### Measurement of saline absorption.

Evans Blue dye was dissolved in normal saline at 0.32 mg/mL, followed by filtration through a 0.2 μm filter. Mice were injected i.p. with dye-containing saline and euthanized at 1 hour afterward. Peritoneal fat tissues were collected, weighed, and then incubated in formamide (Thermo Fisher Scientific) at room temperature for 48 hours to extract Evans Blue dye. Extracted dye was measured by reading absorbance at 620 nm using a Spark microplate reader (TECAN). Extracted dye was quantified by using a standard curve of Evans Blue dye concentration, and calculated as amount of dye (ng) per mg of dry tissue weight.

### Measurement of lactate levels.

Peripheral blood was collected from mice by lancing the tip of the tail using a sterile scalpel blade. Peritoneal fluid was collected from euthanized mice using a micropipette. Samples of blood and peritoneal fluid were directly added to test strips and tested for lactate levels using a Lactate Plus Blood Lactate Measuring Meter (Nova Biomedical).

### Cancer cell implantation assays.

Age-matched mice were randomized into groups and were either administered normal saline or LRS i.p. or left untreated. Seven days afterward, mice were injected i.p. with 1 × 10^6^ RFP-expressing ID8 cells, tGFP-expressing ID8 cells or tGFP-expressing MC-38 cells. Mice were euthanized at 7 days following ID8 cell inoculation and at 2 days following MC-38 cell inoculation. Omental and mesenteric tissues were collected, frozen in Optimal Cutting Temperature Compound (Thermo Fisher Scientific), and cut into 10 μm sections. Because of the high fat content of gonadal fat pads, these tissues were fixed in formalin, embedded in paraffin, and stained with antibody to tGFP (#PA5-22688, Invitrogen). All slides were stained with DAPI and viewed under a Nikon 80i fluorescence microscope. Images were captured by using NIS-Elements software (Nikon). Areas of RFP and tGFP fluorescence within tissue sections were quantified by using ImageJ software (NIH) and evaluated as described in the figure legends.

### IHC analysis.

For histologic analysis, slides of formalin-fixed, paraffin-embedded tissues were stained with H&E. For immunofluorescence staining, slides of frozen tissue sections were fixed in 4% paraformaldehyde for 5 minutes and blocked with 10% goat serum in phosphate-buffered saline (PBS) for 10 minutes. For intracellular staining, 0.2% Triton X-100 was added to the blocking buffer. Afterward, slides were stained with primary antibodies at 4°C for 16 hours, washed 3 times with PBS, and then incubated with fluorochrome-conjugated secondary antibodies for 1 hour. Types and concentrations of antibodies used are listed in Supplemental Table 2. Slides were then washed 3 times with PBS and stained with DAPI. Antibody staining was quantified by using ImageJ software and evaluated as described in the figure legends.

### Flow cytometry and cell sorting.

Mouse peritoneal fat tissues were disaggregated by incubation in HBSS containing calcium and magnesium with collagenase D (1 mg/mL) and DNase I (100 ng/mL) at 37°C for 30 minutes with shaking. Tissues were then passed through 70 μm nylon mesh (BD Biosciences) to further disaggregate tissues and exclude fat cells. Cells were collected by centrifugation at 400*g* for 10 minutes, suspended in PBS containing 1% BSA, and preincubated for 10 minutes with CD16/32 antibody (#101302, BioLegend) to block nonspecific binding of immunoglobulin to Fc receptors. Cells were then incubated for 30 minutes at 4°C with fluorochrome-conjugated antibodies at concentrations listed in Supplemental Table 2. Afterward, cells were washed with PBS, fixed with 4% paraformaldehyde, and acquired. Acquisition and analysis of flow cytometry data were performed using an Accuri C6 Plus flow cytometer equipped with Accuri C6 Plus software (BD Biosciences). Data plots were generated using FlowJo software (FlowJo). Unfixed cells were sorted by using a BD FACsMelody cell sorter (BD Biosciences). The population of viable singlet cells was determined by forward scatter and side scatter and by staining with Viability Dye eFluor 660 dye. For evaluating major lymphoid and myeloid populations, a minimum of 10,000 gated events was analyzed for each sample. For evaluating minor immune cell populations, subpopulations of macrophages, and endothelial cells, all gated events were analyzed. CountBright Beads (Molecular Probes) were added to all samples to determine absolute cell counts in samples.

### Preparation and analysis of cytocentrifuged samples.

CD45^–^PDPN^+^CD140a^–^ cells were sorted from omental tissues using the gating strategy in Supplemental Figure 2B. Sorted cells were resuspended in PBS containing 1% BSA, deposited onto glass slides using a Shandon cytocentrifuge at 300 rpm at 10*g* for 2 minutes, and then fixed with 4% paraformaldehyde. Cells were permeabilized by incubation in 0.2% Triton X-100 in blocking buffer for 10 minutes. Afterward, cells were stained with antibody to WT1 (#ab89901, Abcam) at 4°C for 16 hours, washed 3 times with PBS, and incubated with fluorochrome-conjugated secondary antibody for 1 hour. Slides were then washed 3 times with PBS and stained with DAPI. A minimum of 500 cells were reviewed under fluorescence microscopy to determine the percentage of WT1^+^ cells. The same procedure was used to prepare slides of sorted omental CD11b^int^F4/80^lo^CX3CR1^+^ cells that were stained with antibody to NF-κB p65 (#8242T, Cell SIgnaling Technology). A minimum of 300 cells were reviewed under fluorescence microscopy to determine the percentage of cells with nuclear NF-κB p65. Concentrations of antibodies used are listed in Supplemental Table 2. In other experiments, monocytes/macrophages were sorted based on their CD11b and F4/80 staining patterns and the gating strategy in Figure 3C. Sorted cells were cytocentrifuged at 10*g* for 2 minutes onto slides as described above and fixed in methanol for 5 minutes. Slides were stained with Giemsa solution for 20 minutes, rinsed in deionized water, and then air-dried.

### Chemotaxis assays.

C57BL/6 mice were either left untreated or administered normal saline i.p. and then euthanized at 1 day thereafter. CD45^–^ cells of the stromal vascular fraction were sorted from omental tissues of both groups of mice and were then plated in bottom wells of Boyden chambers (CytoSelect 96-well Cell Migration Assay from Cell Biolabs Inc.) that were coated with Matrigel (Corning) (1 × 10^5^ cells per well). CD115^+^GFP^+^ monocytes were sorted from peripheral blood of *Cx3cr1^+/GFP^* mice and seeded in upper wells (1 × 10^4^ cells per well). Three hours afterward, migrating monocytes were dissociated from the membrane, detected by CyQuant GR Dye (Cell Biolabs Inc.), and quantified by reading fluorescence at 485 nm (excitation)/530 nm (emission) using a Spark microplate reader.

### Ex vivo assay of mesothelial cell exfoliation.

Whole naive mouse omental tissues were excised and each tissue was placed in a 2.0 mL microfuge tube to which an equivalent volume (1.0 mL) of normal saline or LRS was added. Tissues were incubated in parallel for 1 hour at 37°C with shaking at 300 rpm. Control omental tissues were left untreated. Tissues of all groups were then disaggregated as described above and stained with antibodies to CD45, PDPN, and CD140a (#103108, #127408, #135908, BioLegend). CD45^–^PDPN^+^CD140a^–^ cells that were retained in the omentum were quantified by flow cytometry.

### In vitro assay of mesothelial cell stripping.

Human omental mesothelial cells were plated at > 95% confluence in uncoated and Matrigel-coated 24-well plates and were allowed to adhere. Thereafter, culture media were removed, and cells were incubated with 0.5 mL normal saline or LRS at 37°C for 0, 5, 10, 15, 30, and 60 minutes with gentle shaking at 50 rpm. Following incubation, cells that remained adhered to plates were fixed in 4% paraformaldehyde and were stained with crystal violet solution. Adhered cells were quantified by solubilizing crystal violet dye in 10% acetic acid and reading absorbance at 570 nm using a Spark microplate reader. In other experiments, mesothelial cells were plated in 4-well Permanox chamber slides (Sigma-Aldrich) and were then incubated with normal saline or LRS as above. Cells that remained adhered were fixed in 4% paraformaldehyde and permeabilized by incubation in 0.2% Triton X-100 in blocking buffer for 10 minutes. Cells were then stained with ZO-1 antibody (#33-9100, Invitrogen) at 4°C for 16 hours, washed 3 times with PBS, and incubated with fluorochrome-conjugated secondary antibody for 1 hour. Concentrations of antibodies used are listed in Supplemental Table 2. Slides were then washed 3 times with PBS, stained with DAPI, and reviewed under a Nikon 80i fluorescence microscope.

### Antibody array.

Angiogenesis-associated proteins were detected in CD11b^int^F4/80^lo^MHCII^+^CX3CR1^+^ cells by using the Proteome Profiler Mouse Angiogenesis Array (R&D Systems, ARY015). Cells were sorted from omental tissues of untreated and saline-treated mice using the gating strategy in Supplemental Figure 7D. Sorted cells from 5 mice in each group were then pooled. Equivalent numbers of pooled cells from each group (2.5 × 10^5^) were lysed in buffer provided by the manufacturer. Membranes were incubated with cell lysate at 4°C for 16 hours and were then incubated with antibody cocktail and visualized according to manufacturer’s instructions. Signal intensities of spots on membranes were quantified by using ImageJ software (NIH). The background signal intensity of the negative control spot was subtracted from signal intensities of each protein spot. Signal intensities of protein spots were then normalized to the reference spots. An average signal intensity of a given protein was calculated from 2 replicate spots.

### CX3CL1 ELISA.

Mouse omental tissue was weighed and then homogenized in 1 mL PBS containing proteinase and phosphatase inhibitor cocktail (Thermo Fisher Scientific). Cells were then lysed by ultrasonication (3 pulses at 10 seconds per pulse) on ice, and centrifuged at 5,000*g* at 4°C for 5 minutes. Supernatants were collected and assayed for CX3CL1 by using the mouse CX3CL1 ELISA kit (Invitrogen) according to the manufacturer’s instructions.

### Statistics.

Statistical analysis was performed by using GraphPad Prism 9 software (GraphPad Software Inc.). Normality of data distribution in groups was assessed by Shapiro-Wilk test. For animal studies, the use of a minimum of *n* = 5 mice per group was estimated to detect a difference of 50% in cell counts of a given cell population between groups in a 2-sided test at a significance of *P* < 0.05 and with 80% probability. For in vitro and ex vivo assays, a minimum of 3 independent experiments were performed. Unless indicated otherwise, significance of data was assessed by Dunnett’s test or Tukey’s test for multiple comparisons, or by unpaired 2-tailed Student’s *t* test. Data represent mean ± SD. *P* < 0.05 was considered significant.

### Study approval.

Animal studies were performed in accordance with protocols approved by the IACUC of the University of Texas MD Anderson Cancer Center. Studies using human tissue were performed in accordance with protocols approved by the IRB of the University of Texas MD Anderson Cancer Center and the IRB of the University of Chicago. Mesothelial cells were isolated from residual omental tissue specimens that had received full informed consent from human subjects.

## Author contributions

HA designed experiments, performed experiments, acquired data, analyzed data, wrote the manuscript, and edited the manuscript. WL and SYK designed experiments and edited the manuscript. EL provided human mesothelial cells and edited the manuscript. HN conceived the study, designed experiments, acquired data, analyzed data, wrote the manuscript, edited the manuscript, and supervised the study.

## Supplementary Material

Supplemental data

Supporting data values

## Version 1 06/22/2023

Electronic publication

## References

[1] Meza-Perez S, Randall TD (2017). Immunological functions of the omentum. Trends Immunol.

[2] Shipley PG, Cunningham RS (1916). Studies on the absorption from serous cavities: 1. The omentum as a factor in absorption from the peritoneal cavity. Am J Physiol.

[3] Buscher K (2016). Protection from septic peritonitis by rapid neutrophil recruitment through omental high endothelial venules. Nat Commun.

[4] Marcy HO (1902). The omentum as a surgical factor in laparotomy. JAMA.

[5] Morison R (1906). Remarks on some functions of the omentum. Br Med J.

[6] Hagiwara A (1993). Milky spots as the implantation site for malignant cells in peritoneal dissemination in mice. Cancer Res.

[7] Lee W (2019). Neutrophils facilitate ovarian cancer premetastatic niche formation in the omentum. J Exp Med.

[8] Sodek KL (2012). Cell-cell and cell-matrix dynamics in intraperitoneal cancer metastasis. Cancer Metastasis Rev.

[9] Lengyel E (2010). Ovarian cancer development and metastasis. Am J Pathol.

[10] Arie AB (2013). The omentum and omentectomy in epithelial ovarian cancer: a reappraisal: part II-The role of omentectomy in the staging and treatment of apparent early stage epithelial ovarian cancer. Gynecol Oncol.

[11] Chai SW (2021). Partial versus total omentectomy in patients with gastric cancer: a systemic review and meta-analysis. Cancers (Basel).

[12] Atay A, Dilek ON (2021). Is omentectomy necessary in the treatment of benign or malignant abdominal pathologies? A systematic review. World J Gastrointest Surg.

[13] Wang AW (2019). The contribution of the omentum to the outcome from sepsis: an experimental animal study. Shock.

[14] Ariake K (2015). Effect of omentum removal on the risk for postoperative adhesive small bowel obstruction recurrence: a case-control study. Int J Surg.

[15] Yokoyama Y (2012). Is omentectomy mandatory in the operation for ovarian cancer? Preliminary results in a rat study. Eur J Obstet Gynecol Reprod Biol.

[16] McNally L (2015). Does omentectomy in epithelial ovarian cancer affect survival? An analysis of the Surveillance, Epidemiology, and End Results database. Int J Gynecol Cancer.

[17] Kim MC (2011). Comparative study of complete and partial omentectomy in radical subtotal gastrectomy for early gastric cancer. Yonsei Med J.

[18] Nasioudis D (2021). Is there a benefit of performing an omentectomy for clinical stage I high-grade endometrial carcinoma?. Surg Oncol.

[19] Whiteside OJ (2005). Intra-operative peritoneal lavage--who does it and why?. Ann R Coll Surg Engl.

[20] Rodriguez EF (2013). Abdominopelvic washings: A comprehensive review. Cytojournal.

[21] Blumberg N (2018). 0.9% NaCl (normal saline) - Perhaps not so normal after all?. Transfus Apher Sci.

[22] van Westreenen M (1999). Perioperative lavage promotes intraperitoneal adhesion in the rat. Eur Surg Res.

[23] Połubinska A (2006). Time to reconsider saline as the ideal rinsing solution during abdominal surgery. Am J Surg.

[24] Cwalinski J (2015). Normal saline may promote formation of peritoneal adhesions. Int J Clin Exp Med.

[25] Hanly EJ (2005). Abdominal insufflation with CO2 causes peritoneal acidosis independent of systemic pH. J Gastrointest Surg.

[26] Buechler MB (2019). A stromal niche defined by expression of the transcription factor WT1 mediates programming and homeostasis of cavity-resident macrophages. Immunity.

[27] Ghosn EE (2008). CD11b expression distinguishes sequential stages of peritoneal B-1 development. Proc Natl Acad Sci U S A.

[28] Ghosn EE (2010). Two physically, functionally, and developmentally distinct peritoneal macrophage subsets. Proc Natl Acad Sci U S A.

[29] Okabe Y, Medzhitov R (2014). Tissue-specific signals control reversible program of localization and functional polarization of macrophages. Cell.

[30] Geissmann F (2003). Blood monocytes consist of two principal subsets with distinct migratory properties. Immunity.

[31] Jung S (2000). Analysis of fractalkine receptor CX(3)CR1 function by targeted deletion and green fluorescent protein reporter gene insertion. Mol Cell Biol.

[32] Ueda A (1997). Transcriptional regulation of the human monocyte chemoattractant protein-1 gene. Cooperation of two NF-kappaB sites and NF-kappaB/Rel subunit specificity. J Biol Chem.

[33] Khachigian LM (1995). Nuclear factor-kappa B interacts functionally with the platelet-derived growth factor B-chain shear-stress response element in vascular endothelial cells exposed to fluid shear stress. J Clin Invest.

[34] Tong Q (2006). VEGF is upregulated by hypoxia-induced mitogenic factor via the PI-3K/Akt-NF-kappaB signaling pathway. Respir Res.

[35] Zhao W (2011). NF-κB- and AP-1-mediated DNA looping regulates osteopontin transcription in endotoxin-stimulated murine macrophages. J Immunol.

[36] Bazan JF (1997). A new class of membrane-bound chemokine with a CX3C motif. Nature.

[37] Ludwig A (2002). Fractalkine is expressed by smooth muscle cells in response to IFN-gamma and TNF-alpha and is modulated by metalloproteinase activity. J Immunol.

[38] Landsman L (2009). CX3CR1 is required for monocyte homeostasis and atherogenesis by promoting cell survival. Blood.

[39] Getzin T (2018). The chemokine receptor CX_3_CR1 coordinates monocyte recruitment and endothelial regeneration after arterial injury. EMBO Mol Med.

[40] Williams EL (1999). The effect of intravenous lactated Ringer’s solution versus 0.9% sodium chloride solution on serum osmolality in human volunteers. Anesth Analg.

[41] Semler MW (2018). Balanced crystalloids versus saline in critically ill adults. N Engl J Med.

[42] Severs D (2015). A critical appraisal of intravenous fluids: from the physiological basis to clinical evidence. Nephrol Dial Transplant.

[43] Seo WJ (2021). Omentum preservation as an oncologically comparable and surgically superior alternative to total omentectomy during radical gastrectomy for T3-T4 gastric cancer. Surgery.

[44] Kelton JG (1978). Comparison of chemical composition of peritoneal fluid and serum: a method for monitoring dialysis patients and a tool for assessing binding to serum proteins in vivo. Ann Intern Med.

[47] Agnati LF (2017). Homeostasis and the concept of ‘interstitial fluids hierarchy’: Relevance of cerebrospinal fluid sodium concentrations and brain temperature control (Review). Int J Mol Med.

[48] Lee M (2018). Tissue-specific role of CX_3_CR1 expressing immune cells and their relationships with human disease. Immune Netw.

[49] Popovic M (2008). Thrombin-induced expression of endothelial CX3CL1 potentiates monocyte CCL2 production and transendothelial migration. J Leukoc Biol.

[50] Ishida Y (2008). Chemokine receptor CX3CR1 mediates skin wound healing by promoting macrophage and fibroblast accumulation and function. J Immunol.

[51] Bellocq A (1998). Low environmental pH is responsible for the induction of nitric-oxide synthase in macrophages. Evidence for involvement of nuclear factor-kappaB activation. J Biol Chem.

[52] de-Madaria E (2018). Fluid resuscitation with lactated Ringer’s solution vs normal saline in acute pancreatitis: A triple-blind, randomized, controlled trial. United European Gastroenterol J.

[53] Zitek T (2018). Does intravenous lactated ringer’s solution raise serum lactate?. J Emerg Med.

[54] Didwania A (1997). Effect of intravenous lactated Ringer’s solution infusion on the circulating lactate concentration: Part 3. Results of a prospective, randomized, double-blind, placebo-controlled trial. Crit Care Med.

[55] Kenny HA (2007). Use of a novel 3D culture model to elucidate the role of mesothelial cells, fibroblasts and extra-cellular matrices on adhesion and invasion of ovarian cancer cells to the omentum. Int J Cancer.

