# Supplementary material for: Normal saline remodels the omentum and stimulates its receptivity for transcoelomic metastasis
Source: JCI Insight. 2023 Jun 22;8(12):e167336. doi: 10.1172/jci.insight.167336 (PMC10371238; doi:10.1172/jci.insight.167336)
Supplement: Supplemental data [file jciinsight-8-167336-s202.pdf]

Normal saline remodels the omentum and stimulates its receptivity for transcoelomic metastasis

Hironari Akasaka, WonJae Lee, Song Yi Ko, Ernst Lengyel, Honami Naora

SUPPLEMENTAL MATERIALS

Supplemental Figure 1. Effect of normal saline on peritoneal pH.

Supplemental Figure 2. Analysis of mesothelial cells, $CD45^+$ cells, and lymphatic structures.

Supplemental Figure 3. Effect of normal saline on immune cell populations in the omentum.

Supplemental Figure 4. Morphology of omental monocyte/macrophage populations.

Supplemental Figure 5. Effect of normal saline on immune cell populations in mesenteric and gonadal fat tissues.

Supplemental Figure 6. Distribution of Ly6C, CCR2 and CX3CR1 in $CD11b^{Int}F4/80^{Lo}$ subpopulations.

Supplemental Figure 7. Analysis of $Cx3cr1^{+/GFP}$ and $Cx3cr1^{GFP/GFP}$ mice and $CX3CR1^+$ SPM-like cells.

Supplemental Figure 8. Effects of LRS on peritoneal pH and lactate levels.

Supplemental Figure 9. Effect of LRS on immune cell populations and histology of fat tissues.

Supplemental Table 1: Characteristics of body fluids, normal saline and LRS.

Supplemental Table 2. Sources and concentrations of antibodies.

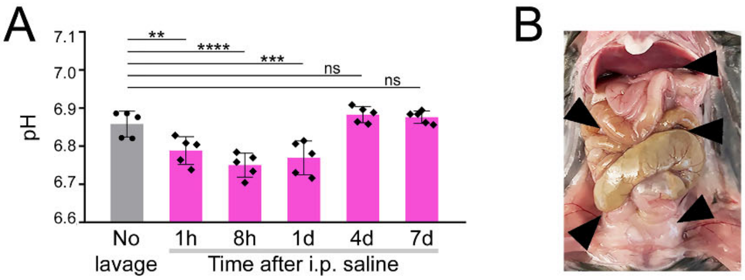

Supplemental Figure 1. Effect of normal saline on peritoneal pH.
(**A**) pH in the peritoneal cavity of adult female C57BL/6 mice at 1 hour and 8 hours, and at 1, 4 and 7 days, following i.p. administration of normal saline (12.5 mL/kg) (n = 5 mice at each time-point). (**B**) For each mouse at a given time-point, an average pH was calculated from measurements taken at five sites indicated by arrowheads. ns, not significant, **$P < 0.01$, ***$P < 0.001$, ****$P < 0.0001$, by Dunnett's multiple comparisons test compared to untreated mice (no lavage) in **A**.

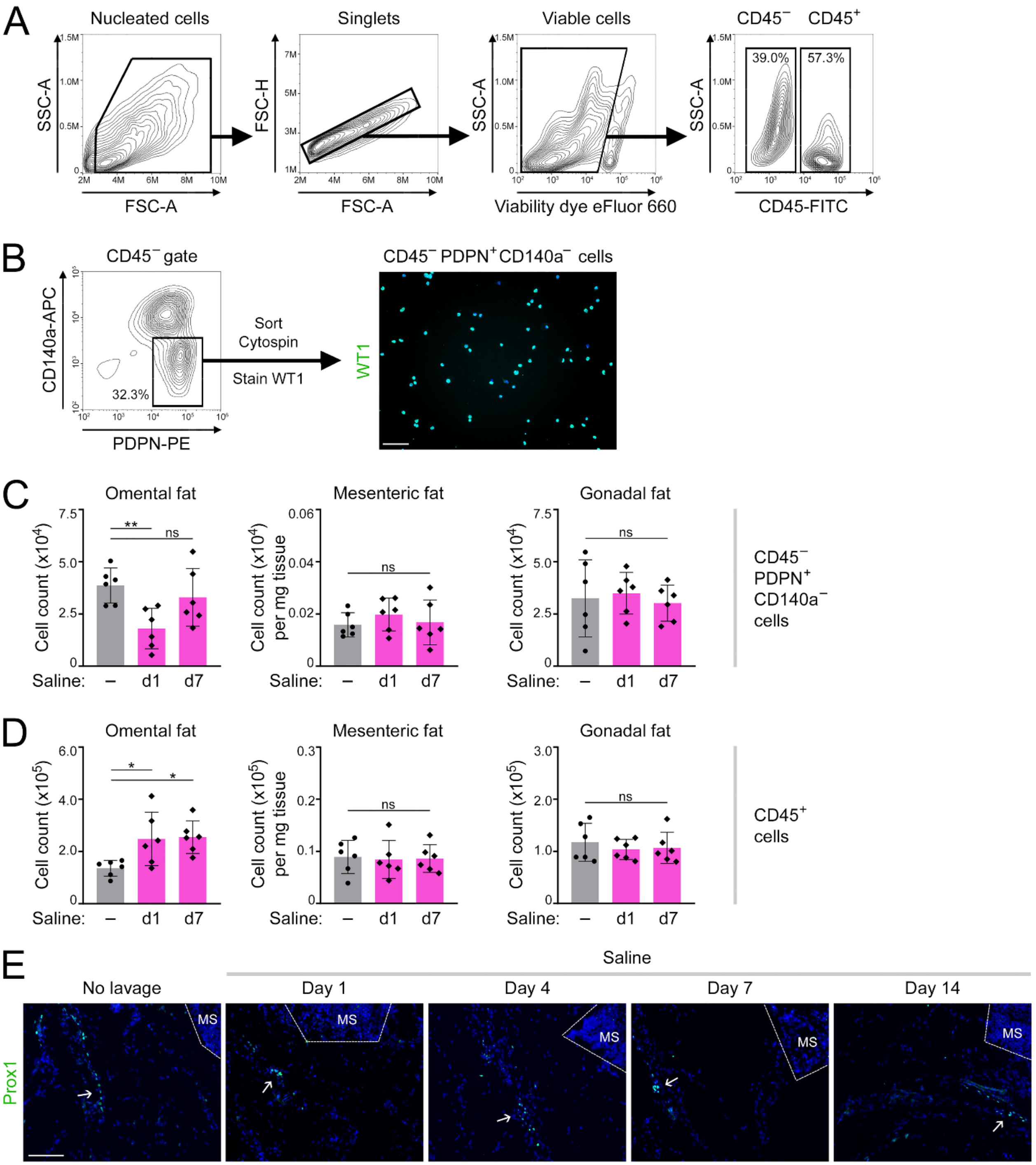

Supplemental Figure 2. Analysis of mesothelial cells, CD45$^+$ cells, and lymphatic structures.
(**A**) Flow cytometry gating strategy for analyzing mouse omentum. (**B**) PDPN and CD140a staining within gated omental CD45$^-$ cells. CD45$^-$ PDPN$^+$CD140a$^-$ cells were sorted, stained for WT1, and counterstained with DAPI. Scale bar, 100 μm. (**C** and **D**) Numbers of CD45$^-$PDPN$^+$CD140a$^-$ cells (**C**) and CD45$^+$ cells (**D**) per omental fat band (greater and lesser omentum combined), per mg of mesenteric fat tissue, and per gonadal fat pad (two lobes combined) in untreated mice and at day 1 and day 7 following i.p. administration of normal saline (n = 6 per group). (**E**) Representative images of staining of Prox1 (denoted by arrows), a master transcriptional regulator of lymphatic development, in the omentum following saline administration. Milky spots (MS) are outlined. Scale bar, 100 μm. Adult female C57BL/6 mice were used in **A**-**E**. *$P < 0.05$, **$P < 0.01$, by Dunnett's multiple comparisons test compared to untreated mice in **C** and **D**.

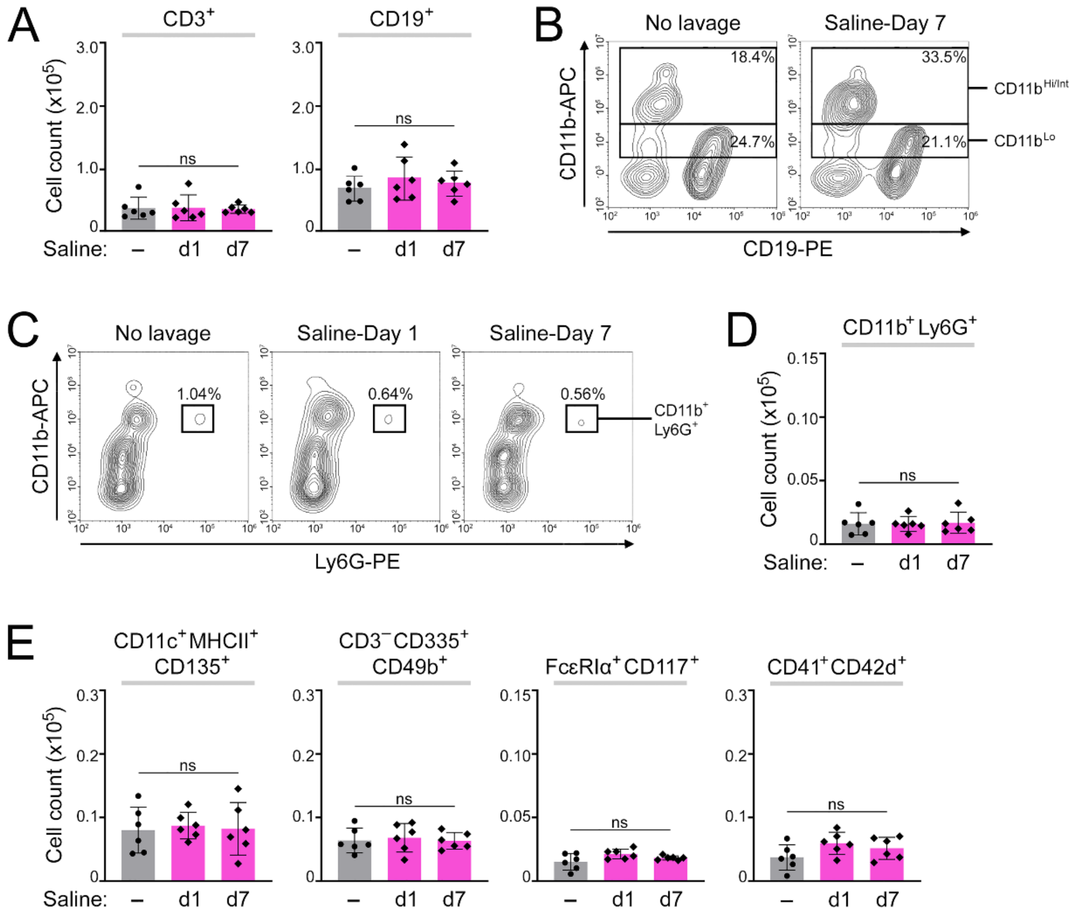

Supplemental Figure 3. Effect of normal saline on immune cell populations in the omentum. Analysis of immune cell populations in omental tissues of untreated mice and at day 1 and day 7 following i.p. administration of normal saline (*n* = 6 per group). Adult female C57BL/6 mice were used. (**A**) Numbers of T cells ($CD3^+$) and B cells ($CD19^+$) per omental fat band. (**B**) Representative plots of CD19 and CD11b staining within gated $CD45^+$ cells. (**C**) Representative plots of CD11b and Ly6G staining within gated $CD45^+$ cells, showing abundance of neutrophils ($CD11b^+Ly6G^+$). (**D**) Numbers of neutrophils per omental fat band. (**E**) Numbers of dendritic cells ($CD11c^+MHCII^+CD135^+$), NK cells ($CD3^-CD335^+CD49b^+$), mast cells ($Fc\varepsilon RI\alpha^+$ $CD117^+$) and platelets ($CD41^+CD42d^+$) per omental fat band. Data in **A, D** and **E** was evaluated by Dunnett's multiple comparisons test compared to untreated mice for each given cell population.

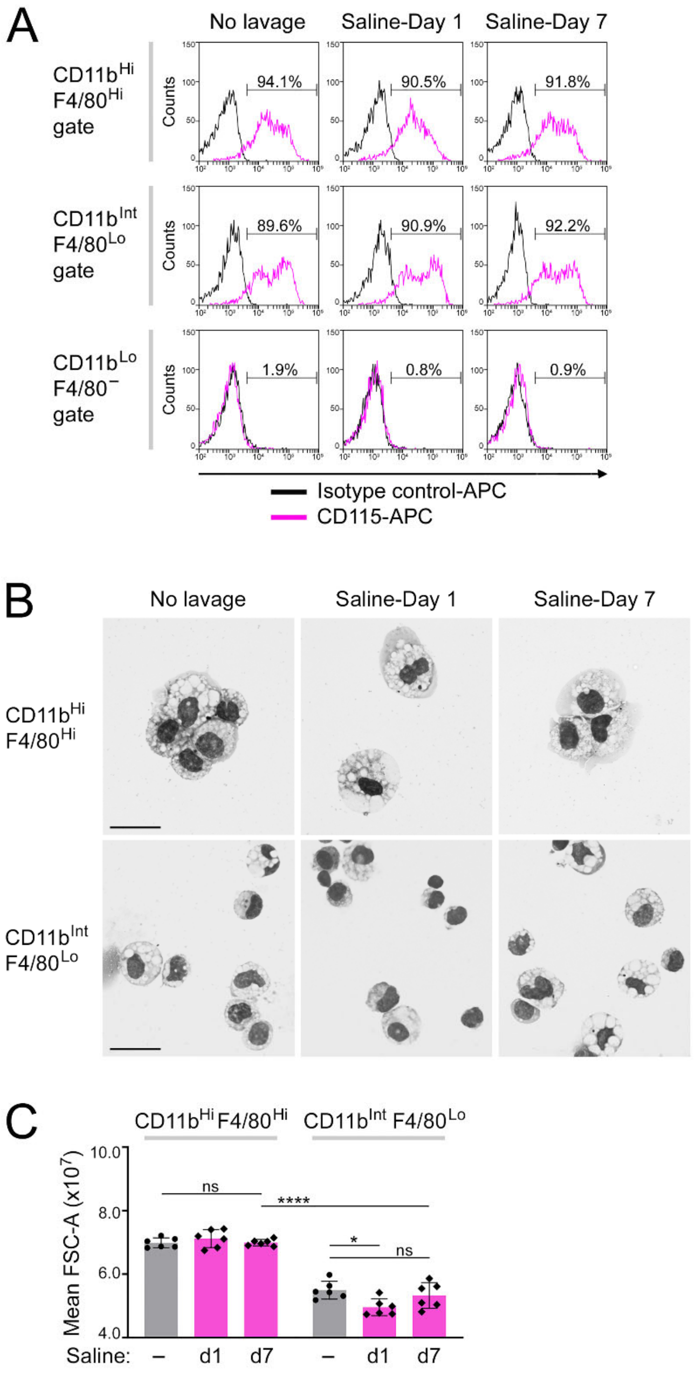

Supplemental Figure 4. Morphology of omental monocyte/macrophage populations.
Analysis of $CD11b^{Hi}F4/80^{Hi}$ and $CD11b^{Int}F4/80^{Lo}$ populations in omental tissues of untreated mice and at day 1 and day 7 following i.p. administration of normal saline. Adult female C57BL/6 mice were used. (**A**) Representative histogram plots of CD115 staining within gated $CD11b^{Hi}F4/80^{Hi}$ and $CD11b^{Int}F4/80^{Lo}$ populations. As a negative control, CD115 staining was evaluated within the gated $CD11b^{Lo}F4/80^{-}$ population that is largely composed of peritoneal B-1 cells (refer Supplemental Figure 3B). (**B**) Morphology of $CD11b^{Hi}$ $F4/80^{Hi}$ and $CD11b^{Int}F4/80^{Lo}$ cells that were sorted from omental tissues and stained with Giemsa solution. Scale bar, 20 µm. (**C**) Relative sizes of $CD11b^{Hi}F4/80^{Hi}$ and $CD11b^{Int}F4/80^{Lo}$ cells, evaluated by forward scatter analysis. Data of $n = 6$ mice per group is shown. *$P < 0.05$, ****$P < 0.0001$, by Tukey's multiple comparisons test in **C**.

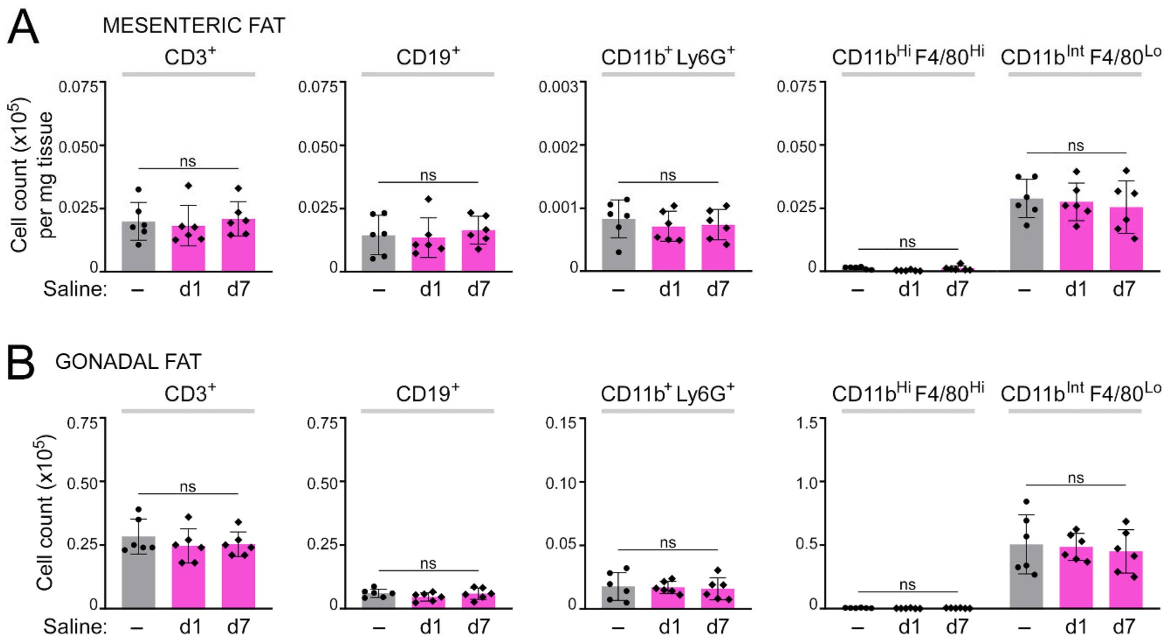

Supplemental Figure 5. Effect of normal saline on immune cell populations in mesenteric and gonadal fat tissues.

T cells ($CD3^+$), B cells ($CD19^+$), neutrophils ($CD11b^+Ly6G^+$) and monocytes/macrophages ($CD11b^{Hi}F4/80^{Hi}$ and $CD11b^{Int}F4/80^{Lo}$) were evaluated in mesenteric and gonadal fat tissues of untreated mice and at day 1 and day 7 following i.p. administration of normal saline (*n* = 6 per group). Adult female C57BL/6 mice were used. (**A**) Numbers of cells in each population per mg of mesenteric fat tissue. (**B**) Numbers of cells in each population per gonadal fat pad (two lobes combined). Data in **A** and **B** was evaluated by Dunnett's multiple comparisons test compared to untreated mice for each given cell population.

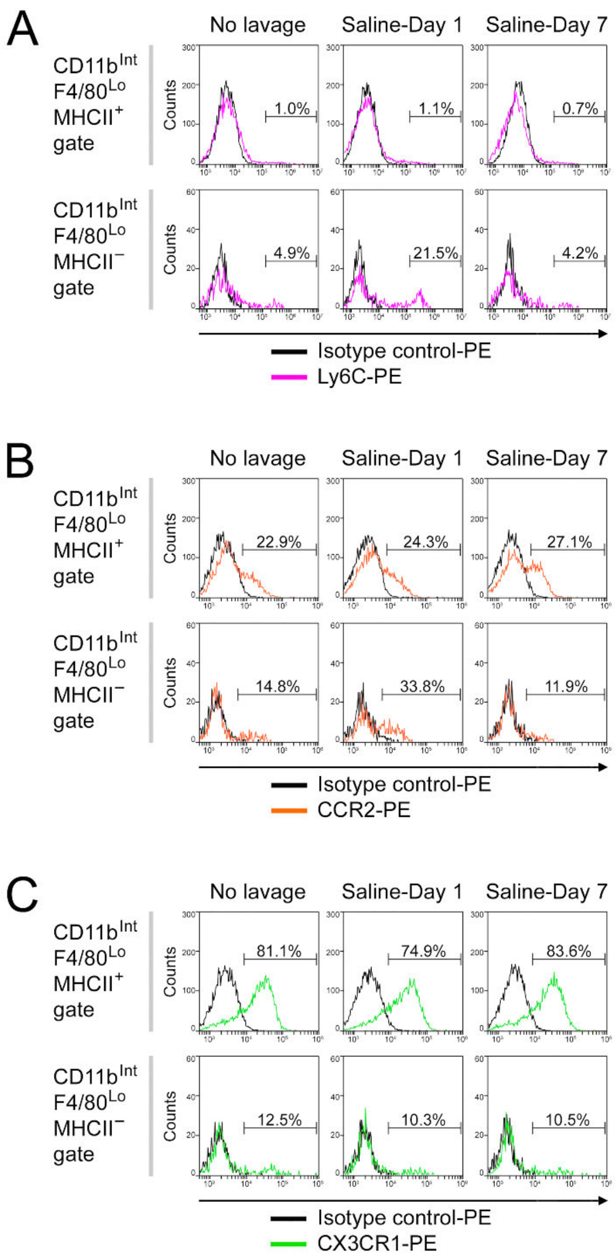

Supplemental Figure 6. Distribution of Ly6C, CCR2 and CX3CR1 in CD11b^{Int}F4/80^{Lo} subpopulations. Representative histogram plots of staining of (**A**) Ly6C, (**B**) CCR2 and (**C**) CX3CR1 within gated CD11b^{Int} F4/80^{Lo}MHCII^{+} and CD11b^{Int}F4/80^{Lo}MHCII^{-} subpopulations in omental tissues of untreated mice and at day 1 and day 7 following i.p. administration of normal saline. Gated populations are shown in Figure 3E. Adult female C57BL/6 mice were used.

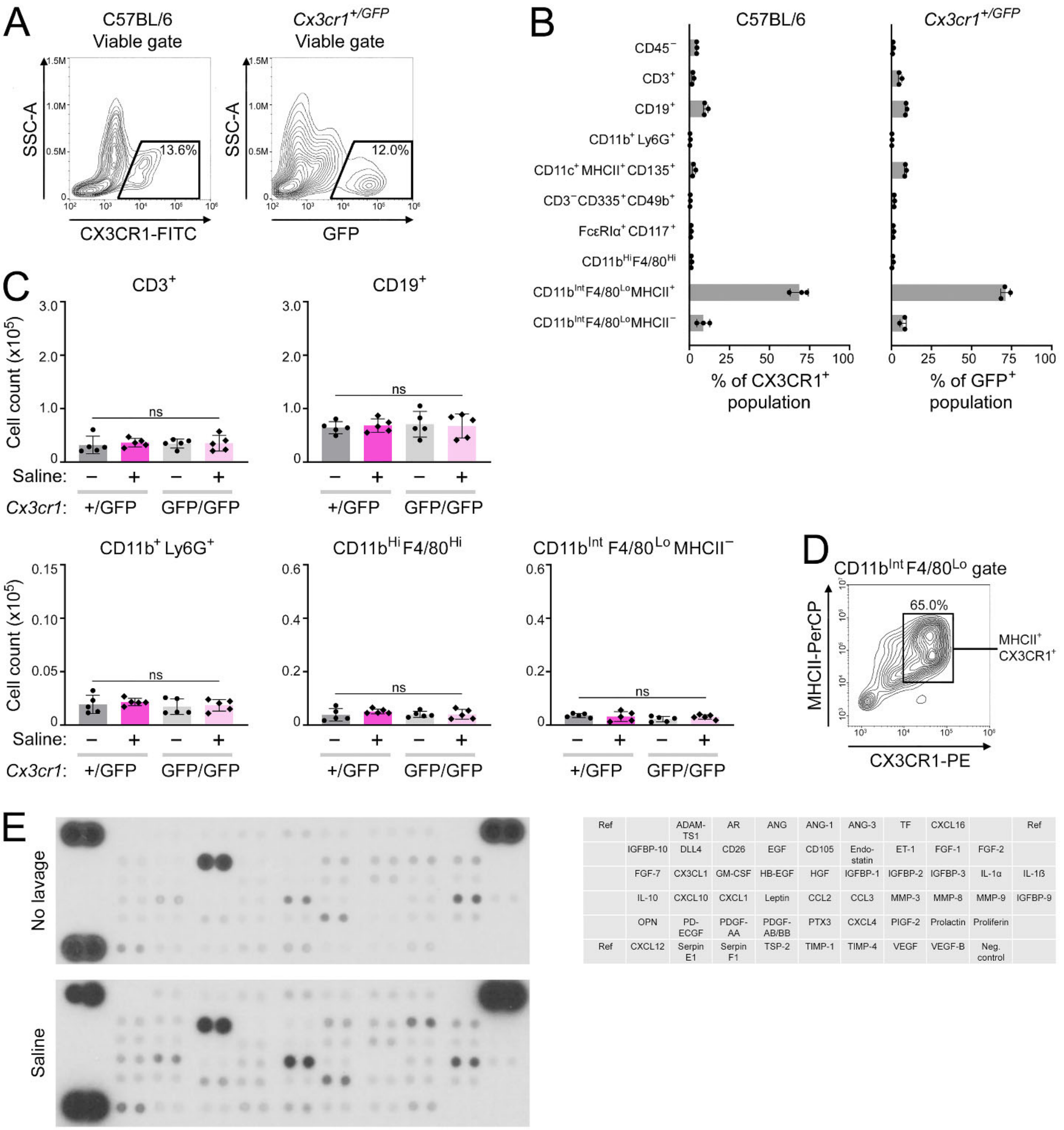

Ref		ADAM-TS1	AR	ANG	ANG-1	ANG-3	TF	CXCL16		Ref
	IGFBP-10	DLL4	CD26	EGF	CD105	Endo-statin	ET-1	FGF-1	FGF-2	
	FGF-7	CX3CL1	GM-CSF	HB-EGF	HGF	IGFBP-1	IGFBP-2	IGFBP-3	IL-1α	IL-1ß
	IL-10	CXCL10	CXCL1	Leptin	CCL2	CCL3	MMP-3	MMP-8	MMP-9	IGFBP-9
	OPN	PD-ECGF	PDGF-AA	PDGF-AB/BB	PTX3	CXCL4	PIGF-2	Prolactin	Proliferin	
Ref	CXCL12	Serpin E1	Serpin F1	TSP-2	TIMP-1	TIMP-4	VEGF	VEGF-B	Neg. control	

Supplemental Figure 7. Analysis of *Cx3cr1*$^{+/GFP}$ and *Cx3cr1*$^{GFP/GFP}$ mice and CX3CR1^{+} SPM-like cells. (**A** and **B**) Comparison of CX3CR1^{+} cells in the omentum of C57BL/6 mice and GFP^{+} cells in the omentum of *Cx3cr1*$^{+/GFP}$ mice. (**A**) Gating strategy. (**B**) Percentages of cell types within the gated CX3CR1^{+} population in C57BL/6 mice (n = 3) and GFP^{+} population in *Cx3cr1*$^{+/GFP}$ mice (n = 3). (**C**) Adult female *Cx3cr1*$^{+/GFP}$ and *Cx3cr1*$^{GFP/GFP}$ mice were left untreated or administered normal saline, and evaluated at 7 days thereafter (n = 5 per group). Shown are cell counts in the indicated immune cell populations per omental fat band. Data was evaluated by Tukey's multiple comparison test. (**D**-**E**) CD11b^{Int}F4/80^{Lo}MHCII^{+}CX3CR1^{+} cells were sorted from omental tissues of adult female C57BL/6 mice that were left untreated and at day 1 following saline administration (n = 5 per group). Sorted cells of each group were then pooled. Lysates of 2.5 x 10^{5} pooled cells of each group were used to probe an antibody array representing 53 angiogenesis-associated proteins. (**D**) Gating strategy showing sorted population. (**E**) Signals detected on array membranes and corresponding proteins (2 replicate spots per protein). Reference (Ref) spots were used for normalization.

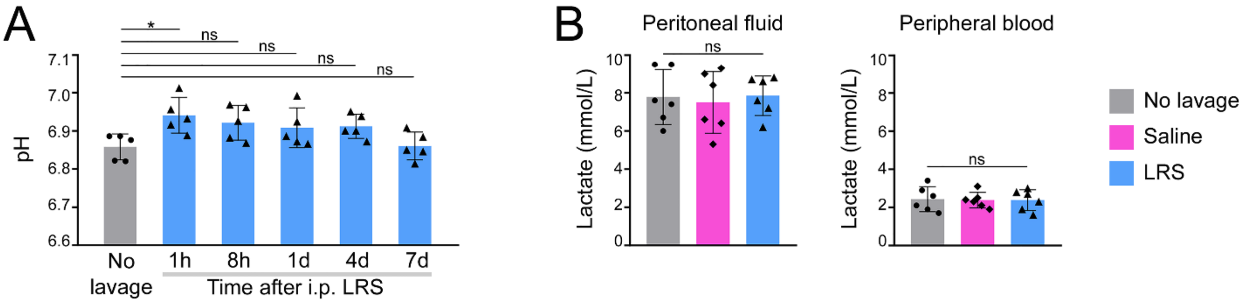

Supplemental Figure 8. Effects of LRS on peritoneal pH and lactate levels.
(**A**) pH in the peritoneal cavity of mice at 1 hour and 8 hours, and at 1, 4 and 7 days, following i.p. administration of LRS (12.5 mL/kg) (n = 5 mice at each time-point). pH measurements were taken as described in Supplemental Figure 1A. Data of untreated mice is duplicated in Supplemental Figure 1A. (**B**) Lactate levels in peritoneal fluid and peripheral blood of untreated mice and at day 1 following i.p. administration of normal saline or LRS at the same dosage (12.5 mL/kg) (n = 6 per group). Adult female C57BL/6 mice were used in **A** and **B**. *P < 0.05, by Dunnett's multiple comparisons test compared to untreated mice (no lavage) in **A** and **B**.

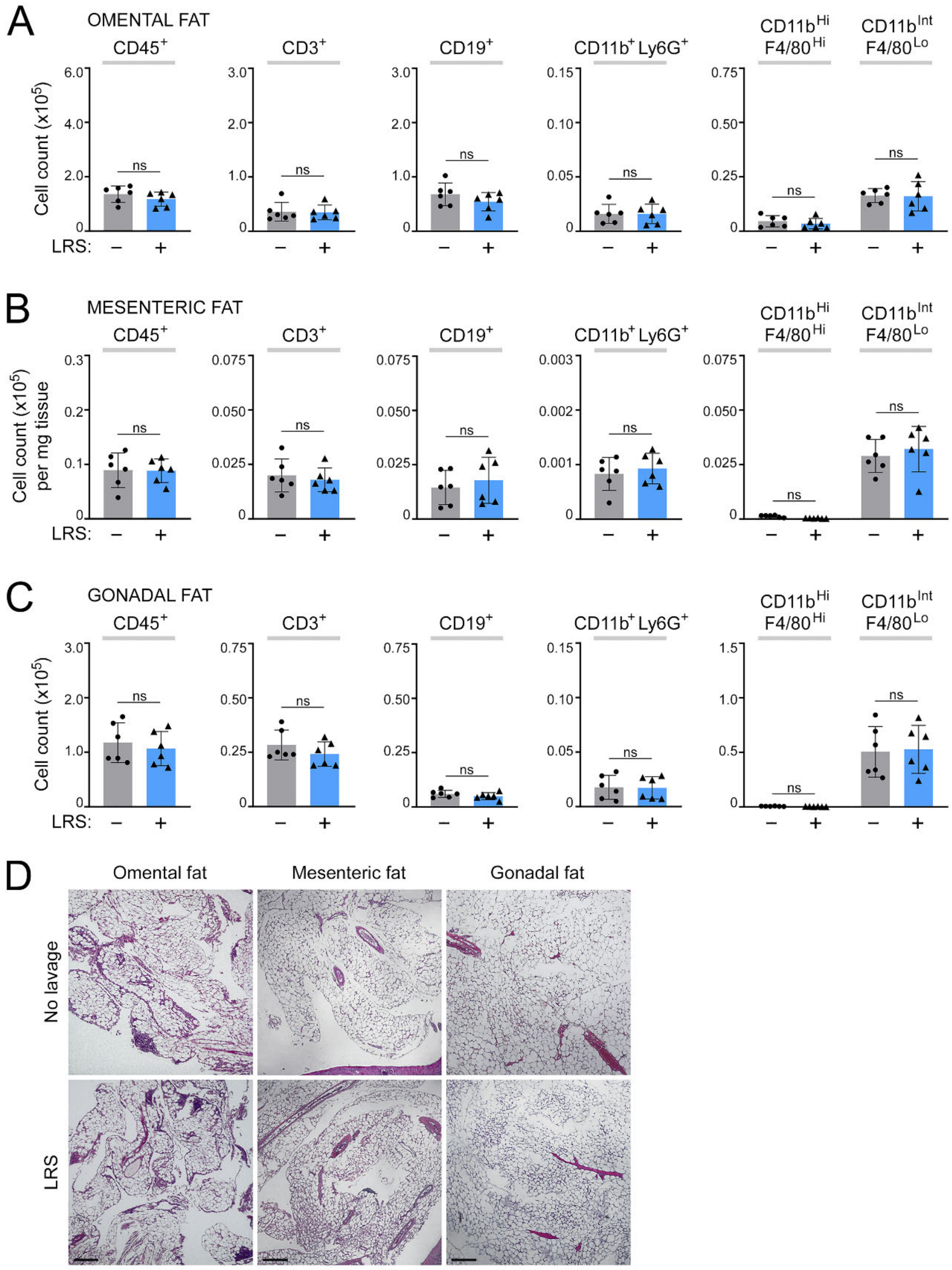

Supplemental Figure 9. Effect of LRS on immune cell populations and histology of fat tissues. (**A**-**C**) Numbers of total immune cells, T cells, B cells, neutrophils and monocytes/macrophages in omental (**A**), mesenteric (**B**), and gonadal (**C**) fat tissues of untreated mice and at day 7 following i.p. administration of LRS (*n* = 6 per group). Data of untreated mice in **A** is duplicated in Supplemental Figure 2D, 3A and 3D, and Figure 3D. Data of untreated mice in **B** and **C** is duplicated in Supplemental Figure 2D, 5A and 5B. (**D**) Representative H&E-stained tissue sections of untreated and LRS-treated mice. Scale bar, 200 µm. Adult female C57BL/6 mice were used in **A**-**D**. Data in **A**-**C** was evaluated by unpaired two-tailed Student’s *t*-test.

Supplemental Table 1:
Characteristics of body fluids, normal saline and LRS*

	Serum/ Plasma	Interstitial fluids	Normal saline (0.9% NaCl)	Lactated Ringer's solution (LRS)
Sodium (mmol/L)	136 - 145	136 - 145	154	130
Chloride (mmol/L)	102 - 108	108 - 118	154	109
Potassium (mmol/L)	4.0 - 5.1	3.5 - 5.0	0	4.0
Calcium (mmol/L)	1.3 - 2.5	1.2 - 2.8	0	2.7
Bicarbonate/Lactate (mmol/L)	20 - 25	19 - 28	0	28
pH	7.38 - 7.42	7.35 - 7.45	5.5	6.5 - 6.75
Osmolarity (mOsmol/L)			308	273
Osmolality (mOsmol/kg)	280 - 296	280 - 296	286	254

*Data compiled from references 21, 42, 44-47.

Supplemental Table 2:
Sources and concentrations of antibodies

**Antibodies for immunohistochemistry and immunocytochemistry**				
**Antibody**	**Clone #**	**Vendor**	**Identifier**	**Concentration**
CD31		Abcam	ab28364	0.065 µg/mL
CD31	RM0032-1D12	Abcam	ab56299	5.0 µg/mL
CD45	I3/2.3	Abcam	ab25386	1.0 µg/mL
CX3CL1		Invitrogen	14-7986-81	1.0 µg/mL
Prox1	Poly19252	BioLegend	925202	1.0 µg/mL
NF-κB p65	D14E12	Cell Signal.Tech.	8242T	0.5 µg/mL
TurboGFP		Invitrogen	PA5-22688	5.0 µg/mL
Wilms Tumor Protein (WT1)	CAN-R9(IHC)-56-2	Abcam	ab89901	4.4 µg/mL
ZO-1	ZO1-1A12	Invitrogen	33-9100	5.0 µg/mL
Alexa Fluor® 488 Goat anti-rabbit IgG (H+L)		Invitrogen	A-11008	2.0 µg/mL
Alexa Fluor® 488 Goat anti-rat IgG (H+L)		Invitrogen	A-11006	2.0 µg/mL
Alexa Fluor® 594 Goat anti-rabbit IgG (H+L)		Invitrogen	A-11012	2.0 µg/mL
Alexa Fluor® 594 Goat anti-rat IgG (H+L)		Invitrogen	A-11007	2.0 µg/mL
**Antibodies for flow cytometry**				
**Antibody**	**Clone #**	**Vendor**	**Identifier**	**Concentration**
FITC-CD45	30-F11	BioLegend	103108	0.5 µg/mL
FITC-F4/80	BM8	BioLegend	123108	5.0 µg/mL
FITC-CX3CR1	SA011F11	BioLegend	149020	5.0 µg/mL
Alexa Fluor® 488-CD102 (ICAM2)	3C4 (MIC2/4)	BioLegend	105609	5.0 µg/mL
Alexa Fluor® 488-PDPN	PMab-1	BioLegend	156208	5.0 µg/mL
PE-PDPN	8.1.1	BioLegend	127408	2.0 µg/mL
PE-F4/80	BM8	BioLegend	123110	1.0 µg/mL
PE-CD19	1D3/CD19	BioLegend	152408	1.0 µg/mL
PE-Ly6G	1A8	BioLegend	127608	1.0 µg/mL
PE-CD135	A2F10	BioLegend	135306	4.0 µg/mL
PE-CD49b	DX5	BioLegend	108908	2.0 µg/mL
PE-FcεRIα	MAR-1	BioLegend	134307	2.0 µg/mL
PE-CD42d	1C2	BioLegend	148504	4.0 µg/mL
PE-Ly6C	HK1.4	BioLegend	128008	2.0 µg/mL
PE-CX3CR1	SA011F11	BioLegend	149006	2.0 µg/mL
PE-CCR2	SA203G11	BioLegend	150610	2.0 µg/mL
PE-CD31	390	BioLegend	102408	2.0 µg/mL
PE-CD115	AFS98	BioLegend	135505	2.0 µg/mL
PE-CD11b	M1/70	BioLegend	101207	2.0 µg/mL
PerCP/Cy5.5-CD3	17A2	BioLegend	100218	2.0 µg/mL
PerCP/Cy5.5-I-A/I-E (MHCII)	M5/114.15.2	BioLegend	107626	0.5 µg/mL
PerCP/Cy5.5-CD45	30-F11	BioLegend	103132	0.2 µg/mL
PerCP/Cy5.5-CD41	MWReg30	BioLegend	133918	2.0 µg/mL
PerCP-CD11b	M1/70	BioLegend	101228	2.0 µg/mL
APC-CD140a	APA5	BioLegend	135908	4.0 µg/mL
APC-CD11b	M1/70	BioLegend	101212	2.0 µg/mL
APC-CD3	17A2	BioLegend	100236	2.0 µg/mL
APC-CD11c	N418	BioLegend	117309	2.0 µg/mL

APC-CD335	29A1.4	BioLegend	137608	4.0 µg/mL
APC-CD117	2B8	BioLegend	105811	4.0 µg/mL
APC-CD115	AFS98	BioLegend	135509	2.0 µg/mL
APC-F4/80	BM8	BioLegend	123116	2.0 µg/mL
APC-CX3CL1	126315	R&D Systems	FAB571A-025	2.5 µg/mL
Purified anti-mouse CD16/32	93	BioLegend	101302	5.0 µg/mL
FITC-Mouse IgG2a isotype control	MOPC-173	BioLegend	400208	5.0 µg/mL
FITC-Rat IgG2a isotype control	RTK2758	BioLegend	400505	5.0 µg/mL
FITC-Rat IgG2b isotype control	RTK4530	BioLegend	400606	0.5 µg/mL
Alexa Fluor® 488- Rat IgG2a isotype control	RTK2758	BioLegend	400525	5.0 µg/mL
PE-Armenian Hamster IgG isotype control	HTK888	BioLegend	400908	2.0-4.0 µg/mL
PE-Syrian Hamster IgG isotype control	SHG-1	BioLegend	402008	2.0 µg/mL
PE-Mouse IgG2a Isotype control	MOPC-173	BioLegend	400212	2.0 µg/mL
PE-Rat IgG2a isotype control	RTK2758	BioLegend	400508	1.0-4.0 µg/mL
PE-Rat IgG2b isotype control	RTK4530	BioLegend	400608	2.0 µg/mL
PE-Rat IgG2c isotype control	RTK4174	BioLegend	400707	2.0 µg/mL
PE-Rat IgM isotype control	RTK2118	BioLegend	400808	2.0 µg/mL
PerCP/Cy5.5-Rat IgG1 isotype control	RTK2071	BioLegend	400426	2.0 µg/mL
PerCP/Cy5.5-Rat IgG2b isotype control	RTK4530	BioLegend	400632	0.2-2.0 µg/mL
APC-Armenian Hamster IgG isotype control	HTK888	BioLegend	400911	2.0 µg/mL
APC-Rat IgG2a isotype control	RTK2758	BioLegend	400511	2.0-4.0 µg/mL
APC-Rat IgG2b isotype control	RTK4530	BioLegend	400612	2.0-4.0 µg/mL
